# Phosphorylation of RGS regulates MAP kinase localization and promotes completion of cytokinesis

**DOI:** 10.26508/lsa.202101245

**Published:** 2022-08-19

**Authors:** William C Simke, Cory P Johnson, Andrew J Hart, Sari Mayhue, P Lucas Craig, Savannah Sojka, Joshua B Kelley

**Affiliations:** 1 Department of Molecular and Biomedical Sciences, University of Maine, Orono, ME, USA; 2 Graduate School of Biomedical Science and Engineering, University of Maine, Orono, ME, USA

## Abstract

Yeast use the G-protein–coupled receptor signaling pathway to detect and track the mating pheromone. The G-protein–coupled receptor pathway is inhibited by the regulator of G-protein signaling (RGS) Sst2 which induces Gα GTPase activity and inactivation of downstream signaling. G-protein signaling activates the MAPK Fus3, which phosphorylates the RGS; however, the role of this modification is unknown. We found that pheromone-induced RGS phosphorylation peaks early; the phospho-state of RGS controls its localization and influences MAPK spatial distribution. Surprisingly, phosphorylation of the RGS promotes completion of cytokinesis before pheromone-induced growth. Completion of cytokinesis in the presence of pheromone is promoted by the kelch-repeat protein, Kel1 and antagonized by the formin Bni1. We found that RGS complexes with Kel1 and prefers the unphosphorylatable RGS mutant. We also found overexpression of unphosphorylatable RGS exacerbates cytokinetic defects, whereas they are rescued by overexpression of Kel1. These data lead us to a model where Kel1 promotes completion of cytokinesis before pheromone-induced polarity but is inhibited by unphosphorylated RGS binding.

## Abstract (precis)

Phosphorylation of the RGS Sst2 alters its subcellular distribution, MAPK localization, and interaction with Kel1, which promotes coordination of polarized growth with completion of cytokinesis.

## Graphical Abstract

**Figure:**
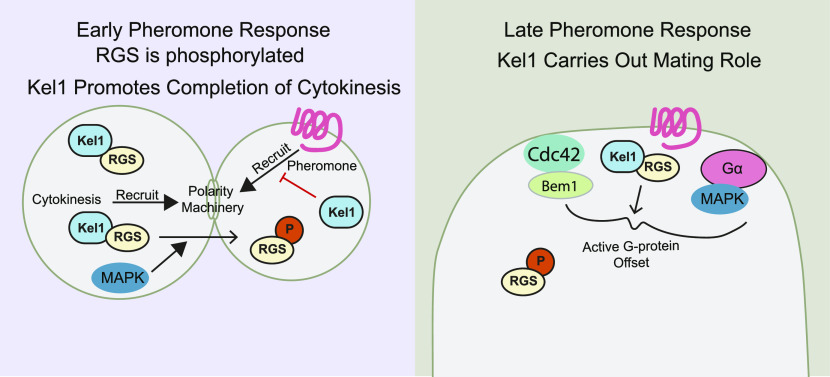

## Introduction

When cells receive multiple signals to perform competing processes, they must integrate those signals to prioritize one outcome. The budding yeast *Saccharomyces cerevisiae* uses a G-protein–coupled receptor (GPCR) to detect and grow toward potential mating partners (Wang & Dohlman, 2004; Arkowitz, 2009; Alvaro & Thorner, 2016). However, the cells must complete mitosis and arrest in G1 before mating projection morphogenesis (shmoo formation) (Peter et al, 1993). This requires that the cell prioritizes the signaling that will drive mitosis and cytokinesis to completion, and only after arrest in G1 can the cell allow the pheromone signaling pathway to commandeer the Cdc42 polarity machinery that has shared uses in both mitosis and pheromone-induced morphogenesis (Park & Bi, 2007; Chiou et al, 2017). Although the mechanism by which G1 arrest occurs is understood, the mechanism responsible for suppression of receptor-driven polarization is unknown.

The pheromone response can be thought of as a response of two G-proteins: the receptor-activated large G-protein consisting of the Gα (Gpa1) and Gβγ (Ste4/Ste18), which conveys information about where the pheromone receptor is active, and the small G-protein Cdc42, which controls actin cytoskeleton polarization and MAPK signaling (Arkowitz, 2009). The GPCR Ste2 activates the large G-protein, causing the Gα and Gβγ subunits to dissociate. Gβγ initiates Cdc42-mediated polarization of the actin cytoskeleton to form a mating projection. Gβγ also promotes the activation of the two yeast ERK homologs Fus3 and Kss1 (Wang & Dohlman, 2004). Of these two MAP kinases, Fus3 has pheromone-specific roles: it is required for gradient tracking, arrest of the cell cycle in G1, and is scaffolded to the cell periphery by active Gα to regulate actin polymerization (Elion et al, 1993; Metodiev et al, 2002; Matheos et al, 2004; Hao et al, 2008; Pope et al, 2014). For this study, we will only be concerned with Fus3 functions, and so all references to MAPK refer to Fus3.

The primary negative regulator of the pheromone pathway is the regulator of G-protein signaling (RGS), Sst2 (Chasse et al, 2006), which serves as the GTPase-activating protein (GAP) for the Gα subunit (Apanovitch et al, 1998). Upon hydrolyzing GTP, the Gα binds to Gβγ, turning off the pathway. RGS function is required for pathway inactivation and for the ability of the cell to track the pheromone gradient (Segall, 1993; Dohlman et al, 1995). GPCR signaling pathways play a central role in human disease, and so the elucidation of RGS signaling mechanisms in *S. cerevisiae* has the potential to inform understanding of human signaling pathways relevant for drug development (Lappano & Maggiolini, 2012; Bar-Shavit et al, 2016; Hauser et al, 2017).

The RGS Sst2 has characterized interactions with the Gα subunit, the pheromone receptor (Ste2), and the MAPK (Fus3) (DiBello et al, 1998; Garrison et al, 1999; Parnell et al, 2005; Ballon et al, 2006). The RGS serves as a GAP for Gα, a function that is enhanced by its binding to the cytoplasmic tail of the receptor (Apanovitch et al, 1998; Ballon et al, 2006; Dixit et al, 2014). The MAPK Fus3 phosphorylates the RGS at serine 539 in a pheromone-dependent manner, but this does not impact the sensitivity of the pathway or downstream MAPK or transcriptional outputs (Garrison et al, 1999; Parnell et al, 2005).

In less well-characterized interactions, the RGS has been found in a yeast two-hybrid screen to interact with the formin Bnr1 and the formin-regulatory protein Kel1 (Burchett et al, 2002; Yu et al, 2008). Kel1 is a kelch-repeat–containing protein which has been shown to act as a negative regulator of Bnr1, is required for efficient mating, and plays a role in the mitotic exit network (MEN) (Hofken & Schiebel, 2002; Gould et al, 2014; Smith & Rose, 2016). Recently, Kel1 has been identified as a noise suppressor in the pheromone pathway (Garcia et al, 2021). We have previously found that the RGS suppresses noise in the pheromone pathway, and so this may indicate a shared function (Dixit et al, 2014). Bnr1 and Kel1 have clear roles in cytokinesis, but the potential for RGS interactions with Bnr1 and Kel1 during the pheromone response has not been pursued.

Here, we set out to determine the role of MAPK phosphorylation of the RGS Sst2 in response to pheromone. We found that RGS phosphorylation is dynamic, with high phosphorylation early in the response, followed by decreased phosphorylation later. Phosphorylation of the RGS decreases its localization to the polar cap and reduces the distance between peak active Cdc42 and peak MAP kinase localization. RGS phosphorylation peaks early in the pheromone response and promotes the completion of cytokinesis before the beginning of pheromone-induced polarity. We find that Kel1 also promotes cytokinetic completion in the presence of pheromone. Improper polarization before cytokinesis is dependent upon the formin Bni1 and suppressed by the formin Bnr1. We found that the RGS Sst2 forms a complex with Kel1 during the pheromone response and that overexpression of Kel1 rescues the cytokinetic defects seen in the unphosphorylatable RGS mutant.

## Results

### Cells track a gradient of pheromone independent of phosphorylation at Ser539

**Figure 1.**
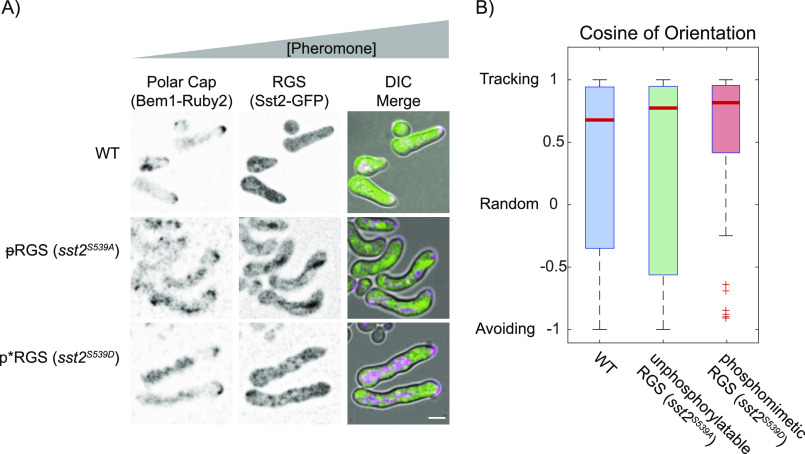
Phosphorylation state of the RGS does not stop gradient tracking. **(A)** Representative live-cell images of WT, unphosphorylatable (ᵽRGS), and phospho-mimetic (p*RGS) RGS expressing the polar-cap marker (Bem1-mRuby2) and the RGS (Sst2-EGFP) tracking a 0–150 nM gradient of pheromone, with pheromone increasing to the right. **(B)** Quantification of gradient tracking cells measured by the cosine of orientation for WT (n = 95), ᵽRGS (*sst2*^*S539A*^, n = 39), and p*RGS (*sst2*^*S539D*^, n = 45) from three experiments. Scale bars represent 5 μm. The differences in gradient tracking were not significant by the pairwise two-sample Kolmogorov–Smirnov test for *P* < 0.05.

The ability to track a gradient of pheromone is dependent upon RGS, specifically its GAP function (Segall, 1993; Dixit et al, 2014). Although previous studies found that GAP activity was not affected by S539 phosphorylation (Garrison et al, 1999), we hypothesized that phosphorylation might change Sst2 function in time or space in a way that affects gradient tracking. We tested this hypothesis by using strains expressing an unphosphorylatable RGS mutant (*sst2*^*S539A*^, denoted ᵽRGS) and phospho-mimetic RGS mutant (*sst2*^*S539D*^, denoted p*RGS) from the endogenous SST2 genomic locus, each fused to GFP. As a marker of the polar cap, we used the Cdc42-GTP–binding protein Bem1, a common polar-cap marker (Kelley et al, 2015), fused to mRuby2. These strains were examined in a microfluidic gradient chamber by live-cell microscopy (Suzuki et al, 2021). We exposed these cells to a 0–150-nM gradient of pheromone and measured their ability to grow toward the source of pheromone (Fig 1A). Both phospho-mutant strains were able to track a gradient of pheromone (Fig 1B). Thus, feedback phosphorylation of RGS has no effect on gradient tracking.

### RGS localization is regulated by its phosphorylation state

**Figure S1.**
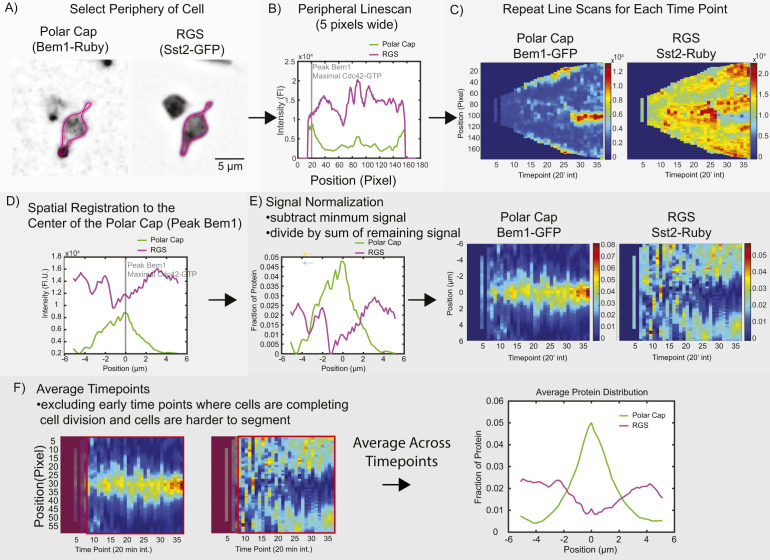
Method for determining protein distribution on the cell periphery. **(A)** The periphery of the cell is defined for a specific time point. **(B)** The intensity of fluorescence signal from each channel is measured with a line width of 5 pixels. **(C)** The periphery for each channel at every time point is measured, here shown as a kymograph. **(D)** The line-scans are adjusted such that the peak intensity of the polar-cap marker Bem1 is set to the center, and the other channel is moved to maintain its original spatial relationship with Bem1. **(E)** The intensity profiles are normalized by subtracting the minimum value and then dividing by the total intensity in the line such that the new line sums to 1. **(F)** For the average distributions, all lines after time point nine are averaged. Time points before this often still include cells completing cytokinesis and are less easily segmented.

Because GAP activity is unchanged, we hypothesized that it may be the spatial distribution of the RGS that is controlled by MAPK phosphorylation. To test this, we again used the p*RGS (*sst2*^*S539D*^) and ᵽRGS (*sst2*^*S539A*^) mutants tagged with EGFP and Bem1-mRuby2 for polar-cap localization. Cells were exposed to saturating pheromone (300 nM) in the microfluidic chamber. To examine the distribution of the RGS along the periphery of the cell, we used our previously reported approach of spatial normalization to the polar cap (Kelley et al, 2015; Shellhammer et al, 2019). Briefly, the signal of RGS along the periphery of the cell is spatially registered to the center of the polar cap as identified by peak Bem1 signal and then averaged to generate a distribution of the protein during the pheromone response (Fig S1) (Kelley et al, 2015). Fluorescence intensity was normalized to sum to 1 so that the values shown indicate the average fraction of protein found at that position relative to the center of the polar cap.

**Figure 2.**
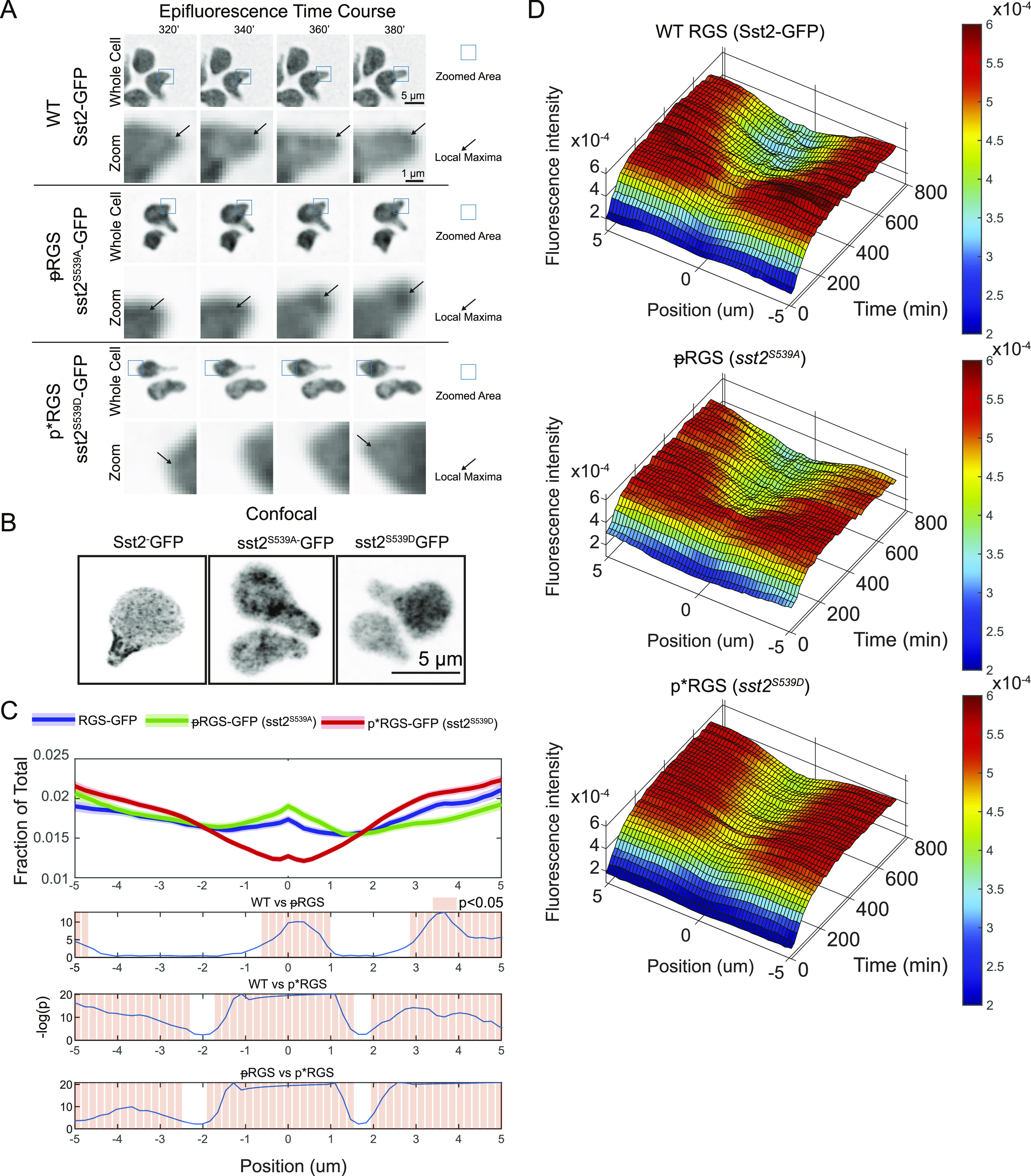
Localization of the RGS is dependent on the phosphorylation state. **(A)** Epifluorescence time course images of the strains expressing the indicated RGS mutants (Sst2-EGFP) imaged in a microfluidic device exposed to 300 nM pheromone for the indicated time. Blue squares indicate area shown enlarged below. Arrows indicated the local maxima of RGS. **(B)** Confocal images of WT, unphosphorylatable (ᵽRGS), and phospho-mimetic (p*RGS) RGS fused to EGFP in saturating pheromone (10 μM). **(C)** Quantification of the average RGS spatial distribution normalized to the polar-cap marker (Bem1-mRuby2) in saturating pheromone over a 12-h time course in a microfluidic gradient chamber, imaged by epifluorescence microscopy. Lines are derived from averaging from 180 min onward. Bottom graphs display statistical analysis using one-way ANOVA followed by Tukey’s honestly significant difference, with −log(*P*-value) plotted in blue, and statistically significant (*P* < 0.05) differences in localization noted by light red bars. Data are derived from n = 89 cells (WT), n = 88 ᵽRGS (unphosphorylatable), and n = 139 p*RGS (phospho-mimetic) cells per strain, with 29 time points per cell. **(A, D)** 3-D kymographs of the spatial distribution of the RGS over 12 h for WT, ᵽRGS, and p*RGS with 37 time points per cell from (A).

We found that WT RGS localizes to both the polar cap and to the periphery of projections where septins would be, consistent with our previous findings (Fig 2A and B) (Dixit et al, 2014; Kelley et al, 2015). Quantitation shows that the phospho-mimetic p*RGS mutation diminishes RGS localization to the polar cap (Fig 2C and D). In contrast, the ᵽRGS mutation leads to a small but statistically significant increase in association with the polar cap. The similarity between the profiles of WT and unphosphorylatable ᵽRGS suggests that much of the RGS measured in WT cells may be in the unphosphorylated form (Fig 2C). When we examined changes in RGS distribution using an averaged 3D-kymograph (Fig 2D), WT and mutant RGS fluorescence increases throughout the time course as expected, which is because of persistent pheromone-induced production of RGS (Dohlman et al, 1996).

### RGS phosphorylation alters the distance between the polar cap and the Gα-MAPK complex

We hypothesized that the phospho-dependent changes in RGS localization would lead to corresponding changes in the localization of active Gα. Although Gα is localized across the membrane (Wang et al, 2005), its localization alone does not indicate the activation state. However, active Gα is known to recruit active MAPK (Metodiev et al, 2002), forming a Gα-MAPK complex that activates the formin Bni1 and promotes gradient tracking (Metodiev et al, 2002; Matheos et al, 2004; Errede et al, 2015). We therefore hypothesized that we could monitor the localization of the Gα-MAPK complex as a proxy for the activation state of Gα.

**Figure 3.**
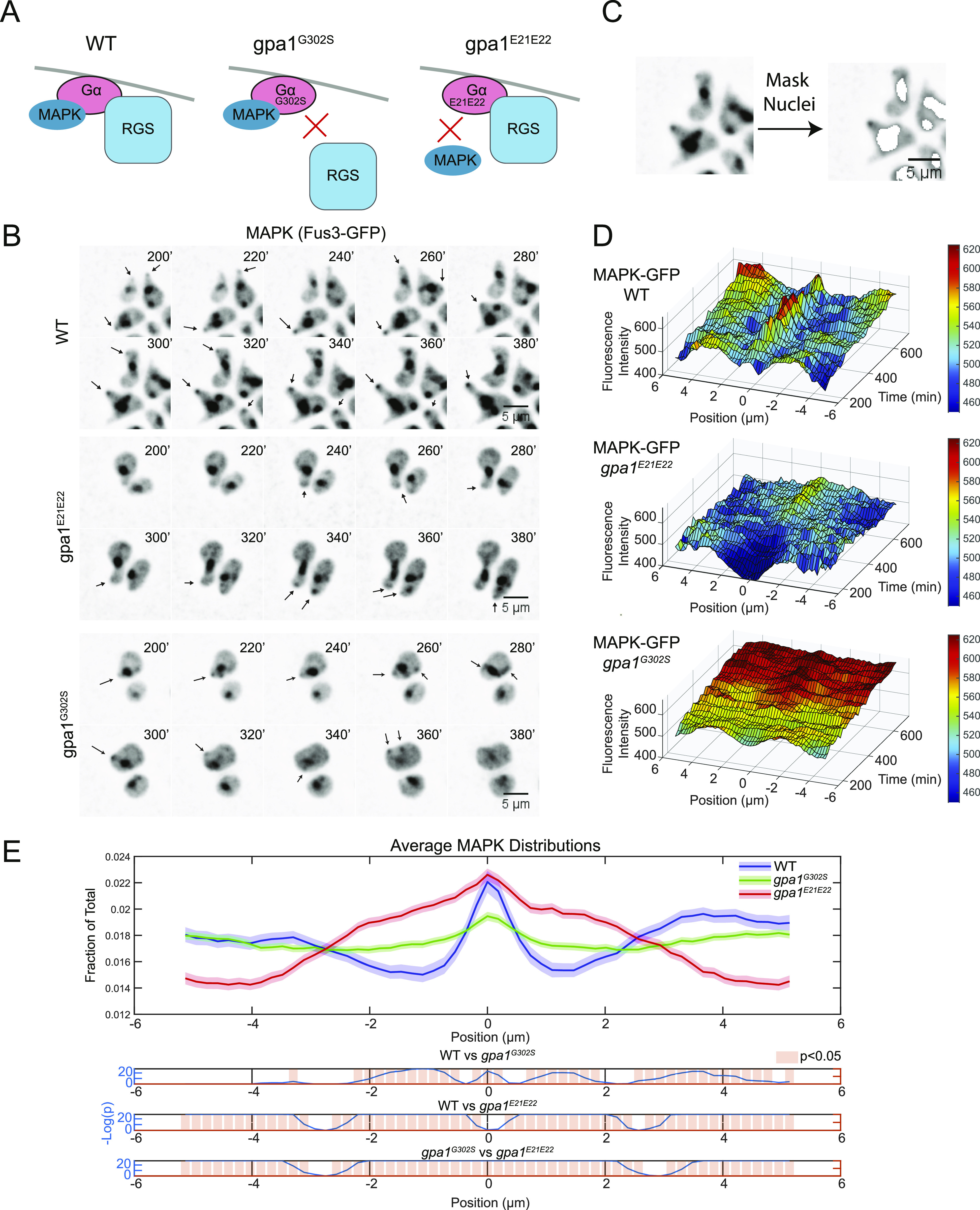
The localization of the MAPK Fus3 is influenced by binding of active Gα. **(A)** Diagram of the effect of the hyperactive *gpa1*^*G302S*^ mutant and the MAPK-uncoupling *gpa1*^*E21E22*^ mutants. **(B)** Epifluorescence time course images of Fus3-GFP with the indicated Gα mutants. Cells were imaged in a microfluidic device for the indicated time and exposed to a flat 300 nM pheromone concentration. **(C)** To quantify peripheral MAPK, the nuclear signal was masked for each cell before quantitation (Shown in Fig S2). **(B, D)** Average kymographs of MAPK localization in the indicated cell line from (B). **(E)** Quantification of the amount of MAPK on the periphery of the cell spatially normalized to the center of the polar cap as in Fig 2. Shaded areas represent 95% confidence intervals. Data are derived from n = 51 (WT), n = 157 (*gpa1*^*G302S*^), and n = 86 (*gpa1*^*E21E22*^), with 29 time points per cell. Bottom graphs display statistical analysis using one-way ANOVA followed by Tukey’s honestly significant difference, with −log(*P*-value) plotted in blue and statistically significant (*P* < 0.05) differences in localization noted by light red bars.

**Figure S2.**
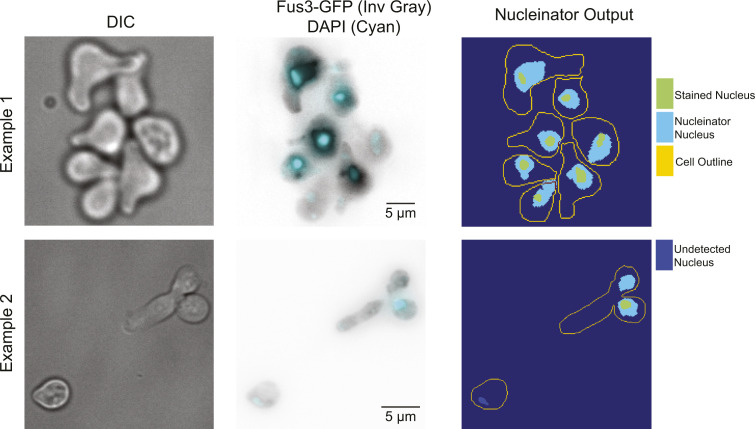
Validation of Nucleinator method for removing nuclear MAPK fluorescence. The Nucleinator algorithm calculates the mean and SD of Fus3-GFP in each cell and selects pixels which are above 1 SD above the mean and are part of a contiguous object that is larger than 25 pixels. Here, we test the ability of the algorithm to detect nuclear pixels by treating cells expressing Fus3-GFP with pheromone followed by fixation and nuclear staining with Hoechst dye. Shown are examples of DIC images, inverted grayscale images of Fus3-GFP, and Hoechst stain in cyan. At the right are the cell outlines with the Nucleinator-detected nuclei and the actual nuclei (either successfully detected, above, or missed, below). Missed nuclei generally appear to be dim in both the Fus3-GFP image and in the Hoechst stain. Over 77 cells, 86.77% of Hoechst defined pixels were identified based on Nucleinator analysis of Fus3-GFP signal.

To test the use of MAPK as a marker for active Gα, we examined a GFP-tagged MAPK (Fus3-GFP) in wild-type cells and two mutants with opposing effects: a Gα mutant that is hyperactive because it no longer interacts with the RGS, *gpa1*^*G302S*^ (DiBello et al, 1998) and a Gα mutant that does not bind MAPK, *gpa1*^*E21E22*^ (Metodiev et al, 2002) (Fig 3A). In all cells, MAPK localizes to the nucleus and to the polar cap (Fig 3B). In the *gpa1*^*E21E22*^ mutant that cannot bind MAPK, there was a marked decrease in association with the polar cap, although at later time points, it appeared at the polar cap more often, potentially through an interaction with a different binding partner (Fig 3D). In the hyperactive *gpa1*^*G302S*^ mutant, MAPK levels consistently increased, and there were often multiple discernible spots of MAPK accumulation (Fig 3B). We assessed MAPK levels along the periphery of the cell in these strains using the same techniques as above, with the exception that the nuclei were masked to exclude nuclear signal from our measurements of the periphery (Fig 3C). We found that if the quantitation was performed with the nuclear signal present, the nuclei were frequently close enough to the periphery of the cell to create spikes in signal ∼1–2 μm from the center of the polar cap. To solve this problem, nuclear masks were generated by using single-cell histogram analysis of the Fus3-GFP signal and removing large objects that were more than 1 SD above the mean, an adaptation of an algorithm we designed to detect nuclear granules (Fig 3C) (Hunn et al, 2022). With this approach, we were able to use the Fus3-GFP signal to identify 86.7% of nuclear pixels from a control with the nucleus marked by Hoechst stain (Fig S2). When examining the shapes of the protein distributions, WT cells have the sharpest distribution of MAPK with respect to the location of the polar cap. Both loss of MAPK binding and excess binding of MAPK broadened its distribution (Fig 3E). We believe the distribution of signal in the Gα MAPK–binding mutant (*gpa1*^*E21E22*^) likely represents the profile of the remaining MAPK-binding partners at the polar cap (Fig 3D). Fus3 binds to the MAPK scaffold Ste5, and the polar cap is populated with many MAPK substrates (Elion et al, 1993; Wang & Dohlman, 2004). We conclude that MAPK localization is measurably affected by binding to active Gα.

Having determined that MAPK localization is affected by its association with active Gα and that RGS localization is altered by phosphorylation, we next examined whether phosphorylation of the RGS affects the distribution of the MAPK, which is predominantly associated with Gα present at the periphery (Fig 3D). We generated strains expressing fluorescent protein fusions of the polar-cap marker (Bem1-mRuby2) and MAPK (Fus3-EGFP) in the presence of the phospho-mimetic and unphosphorylatable RGS mutants. We then imaged those strains in a microfluidic device in the presence of 300 nM pheromone for 12 h, as above.

**Figure 4.**
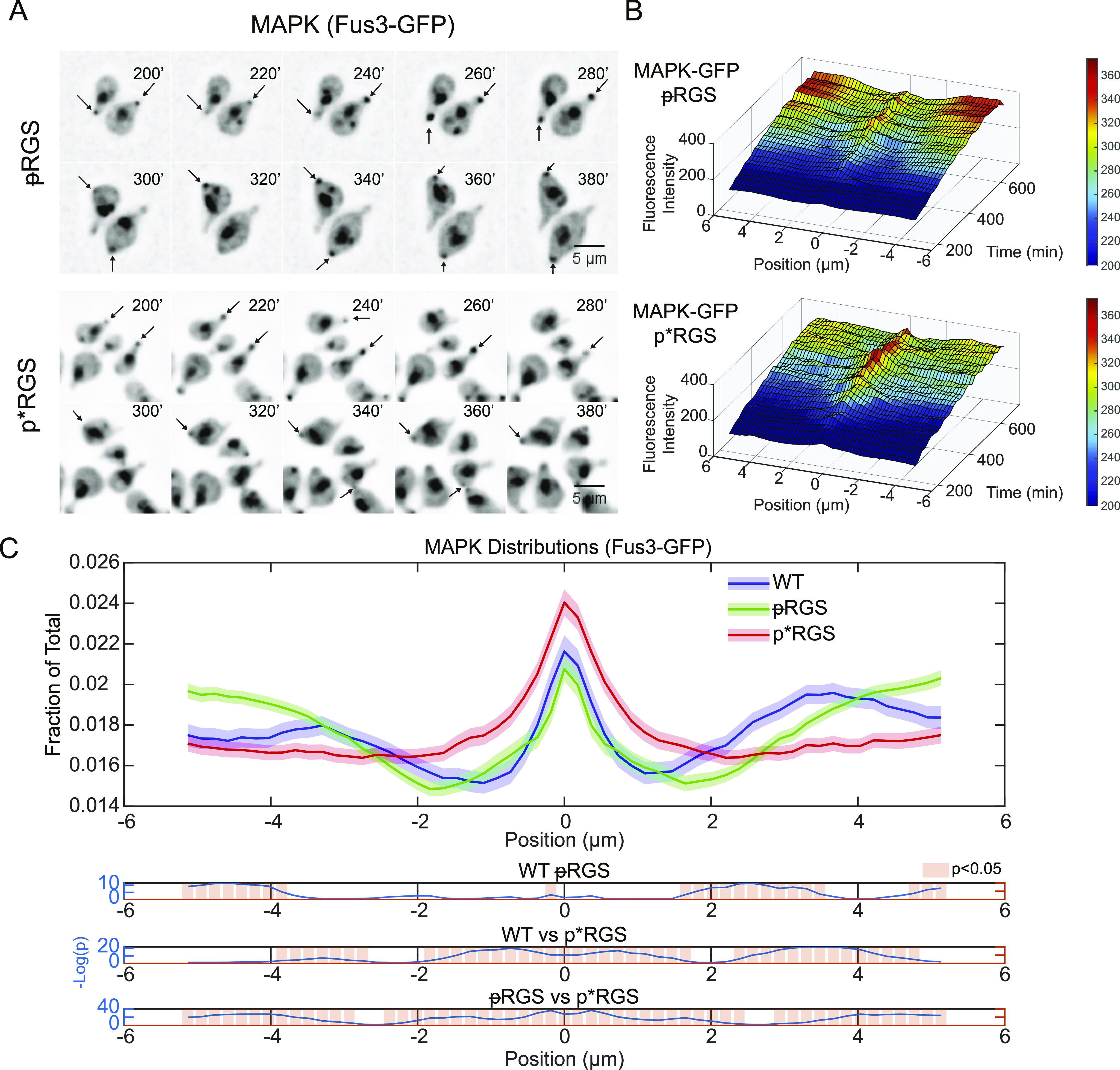
RGS phosphorylation increase MAPK complex levels at the centre of the polar cap. **(A)** Epifluorescence time course images of the MAPK Fus3-GFP with the indicated RGS phospho-mutants. Cells were imaged in a microfluidic device for the indicated time and exposed to a flat 300 nM pheromone concentration. Arrows indicate MAPK associated with the site of polarized growth. **(A, B)** Average kymographs of MAPK localization in the indicated cell line shown in (A). **(C)** Average protein distribution profiles of MAPK (Fus3-GFP) in cells expressing the unphosphorylatable ᵽRGS (*sst2*^*S539A*^) or the phospho-mimetic p*RGS (*sst2*^*S539D*^) aligned to the center of the polar cap (Bem1) as described in Fig 2. Shaded areas represent 95% confidence intervals. Bottom graphs display statistical analysis using one-way ANOVA followed by Tukey’s honestly significant difference, with −log(*P*-value) plotted in blue and statistically significant (*P* < 0.05) differences in localization noted by light red bars. Data are derived from n = 89 (ᵽRGS) and n = 73 (p*RGS) cells and 29 time points per cell.

**Figure 5.**
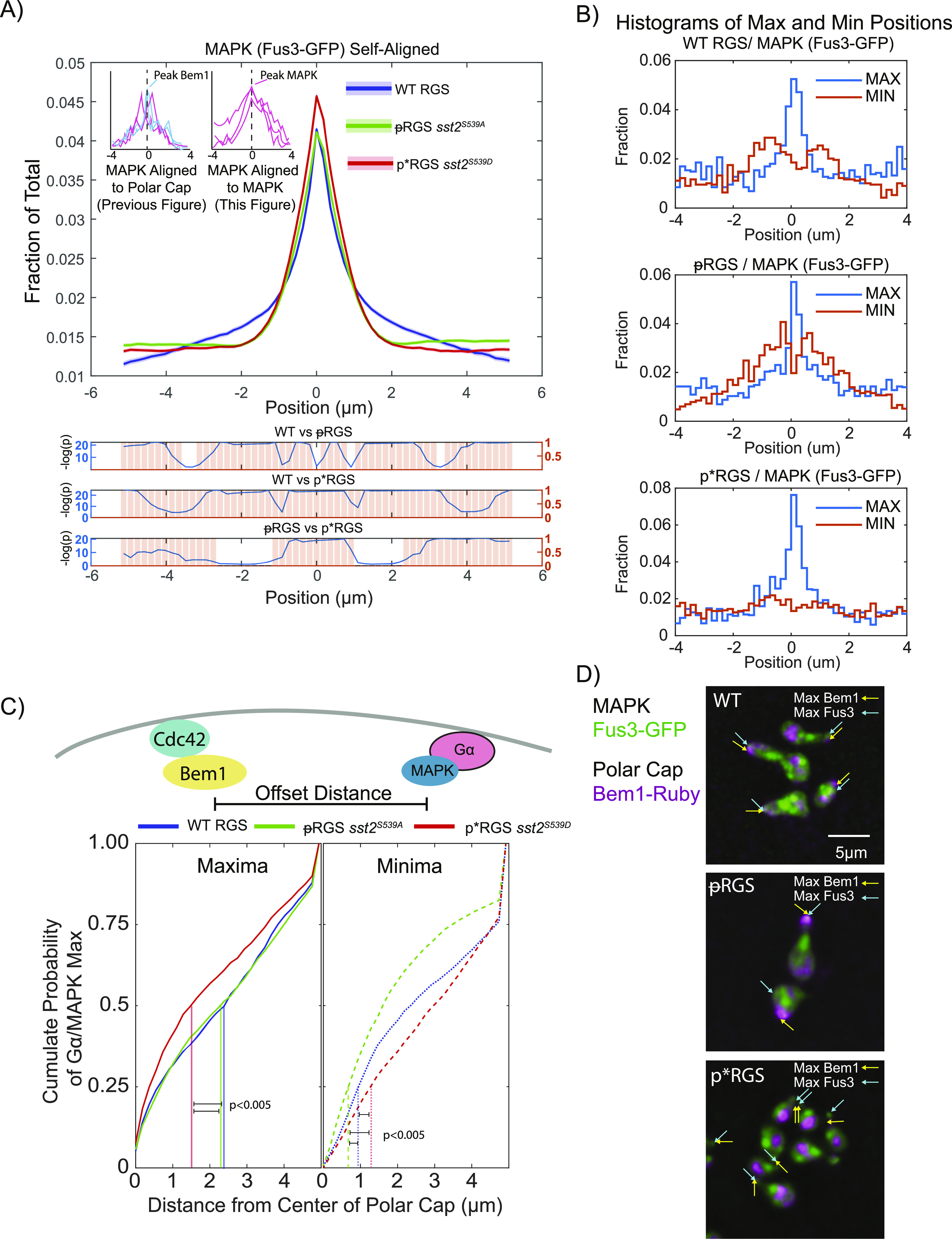
RGS-induced changes in MAPK distribution. **(A)** Distribution of MAPK (Fus3-GFP) from Fig 4, spatially normalized to peak MAPK (Fus3) rather than to the polar cap (Bem1). Shaded areas represent 95% confidence intervals. Statistical analysis was performed as in Fig 2 and is shown in graphs below. **(B)** Histograms of the location of maxima and minima of MAPK spatially registered to the polar cap in the indicated strain. **(C)** Comparison of the distance between the maxima of the Bem1 and MAPK, which bind active Cdc42 and active Gα, respectively. Graphed is the cumulative sum of MAPK maxima (left) and minima (right) versus distance from the polar cap. Vertical lines show the distance where 50% of maxima have appeared and where 25% of minima have appeared. Statistical significance was evaluated by pairwise two-sample Kolmogorov–Smirnov tests. **(D)** Examples of the offset between maximum Bem1 and maximum Fus3 intensity with the indicated RGS mutants.

In both RGS mutants, the presence of MAPK at the cell periphery was much more consistent than we observed in WT cells (Fig 3B versus 4A). This consistently high signal is evident in the decreased noise in the kymographs (Figs 3D and 4B). We see that the unphosphorylatable ᵽRGS mutant (*sst2*^*S539A*^) shows a distinct peak at the center with a local minimum peripheral to the peak (Fig 4B and C). These local minima are also readily visible in the average distributions (Fig 4C), where the unphosphorylatable ᵽRGS mutant (*sst2*^*S539A*^) displays a very similar average MAPK distribution to WT (Fig 5C) with a peak at the center and local minima 1–2 μm from the center. The p*RGS (*sst2*^*S539D*^), however, displayed an enrichment of MAPK at the polar cap (Fig 4C) and decreases steadily toward the edge without the local minima next to the peak seen in the WT and ᵽRGS (Fig 4B and C).

There are two potential explanations for these changes in MAPK distribution: (1) a change in the absolute distribution of MAPK at individual times or (2) MAPK distribution is unchanged but has a different spatial relationship with the polar cap. To assess these possibilities, we examined the distribution of MAPK (Fus3-GFP) spatially normalized to itself instead of Bem1 (Fig 5A). This shows the shape of MAPK localization at any given time in the cell. We see very little difference in the distribution of MAPK in both phospho-mutants when compared with WT RGS (Fig 5A). Although the changes are statistically significant, they are not large enough to account for the changes in the MAPK localization relative to the polar cap seen in Fig 4. If MAPK has the same shape within the cell under these different conditions, then the offset of this shape is changing relative to the polar cap (possibility two above). To test this, we examined the distribution of the maximum and minimum MAPK intensity relative to maximal polar cap intensity (Fig 5B). The localization of the maxima appears to recapitulate the average distributions in Fig 4C, with the phospho-mimetic p*RGS leading to much more frequent MAPK localization to the polar cap. Perhaps more surprising is the distribution of the minima. In WT cells, the most common place to have a minimum intensity of MAPK is immediately proximal to the center of the polar cap, peaking ∼1 μm away (Fig 5B). In the unphosphorylatable ᵽRGS mutant, the minima are again proximal to the center of the polar cap; however, they are closer than in WT, peaking at 0.3–0.5 μm from the center. In the phospho-mimetic p*RGS cells, the minima appear to be much more evenly distributed across the membrane. This suggests an RGS-dependent negative feedback to MAPK proximal to the polar cap that is disrupted by the phosphorylation of the RGS.

We then plotted the cumulative distribution of the distance of maxima and minima from the polar cap. This is effectively measuring the offset along the membrane between Bem1 and Fus3, which bind, respectively, active Cdc42 and active Gα. We find that 50% of MAPK maxima for WT and ᵽRGS fall within ∼2.3 μm of the polar-cap peak, whereas 50% of MAPK maxima in the p*RGS fall within ∼1.5 μm of the polar-cap peak (*P*-values calculated using pairwise Kolmogorov–Smirnov tests) (Fig 5C). We have provided some example images of the localizations of the maximum Bem1 and Fus3 in Fig 5D. In examining where there is a large offset between the polar cap and MAPK, it most often appears in those situations where the MAPK intensity at the polar cap is low, and therefore, other sites along the periphery may be maximal without a significant accumulation of MAPK. Thus, unphosphorylated RGS drives a greater distance between the polar cap and MAPK and based on their binding partners, active Gα and active Cdc42.

When examining the minima (Fig 5C) in the unphosphorylatable ᵽRGS, we have drawn attention to the 25^th^ percentile mark, as ∼25% of minima in WT occur within 1 μm, corresponding to the WT peak of minima identified in Fig 5B. In the unphosphorylatable ᵽRGS mutant, 25% of minima occur within ∼0.6 μm, whereas in the phospho-mimetic p*RGS, 25% of minima occur within 1.2 μm. The difference in the distributions of the minima is statistically significant between all three strains and recapitulates our summary of Fig 5B. We conclude that the phosphorylation of the RGS likely disrupts a negative feedback event targeted proximal to the site of polarity.

### RGS phosphorylation peaks early in the pheromone response and diminishes at later time points

**Figure S3.**
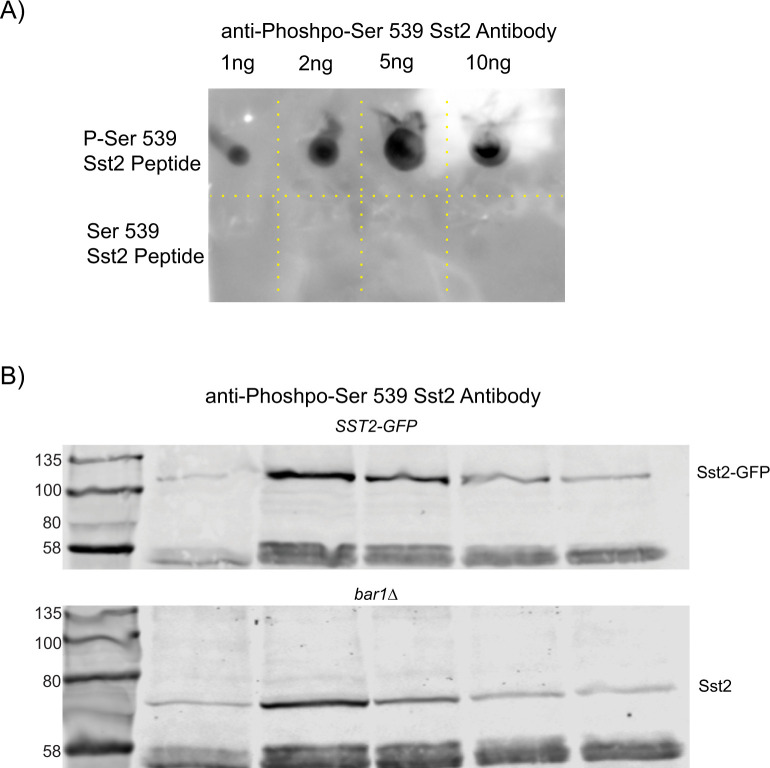
Characterization of the phospho-Ser539-Sst2 antibody. **(A)** Dot blot with the indicated quantities of peptide. The antibody is specific for phospho-peptide under the conditions used. All blotting is carried out with excess unphosphorylated peptides to stop nonspecific binding. **(B)** Our anti-phospo-Sst2 antibody detects a higher molecular weight band when detecting Sst2-GFP than when detecting native Sst2. In addition, the time-dependent decrease in phospho-Sst2 is not dependent upon Bar1-mediated degradation of pheromone.

Previous characterization of the phosphorylation of Sst2 at serine 539 showed Fus3-dependent phosphorylation at 1 h of pheromone treatment (Garrison et al, 1999). Our results suggest that much of the RGS we are quantifying is unphosphorylated RGS as the ᵽRGS mutant routinely looks more like WT than the p*RGS. This led us to hypothesize that Sst2 phosphorylation at S539 may be dynamic: peaking earlier in the response and decreasing at later times. To test the dynamics of phosphorylation, we developed a rabbit polyclonal antibody to detect Sst2 phosphorylated on serine 539, LHPH**S**PLSEC, where the bold serine is phosphorylated. Western blotting of Sst2-GFP versus untagged Sst2 shows a GFP-dependent size shift in the detected band, indicating that the antibody is specific for Sst2 (Fig S3). Western blotting is done in the presence of excess unphosphorylated peptide to ensure that it is specific for the phospho-epitope.

**Figure 6.**
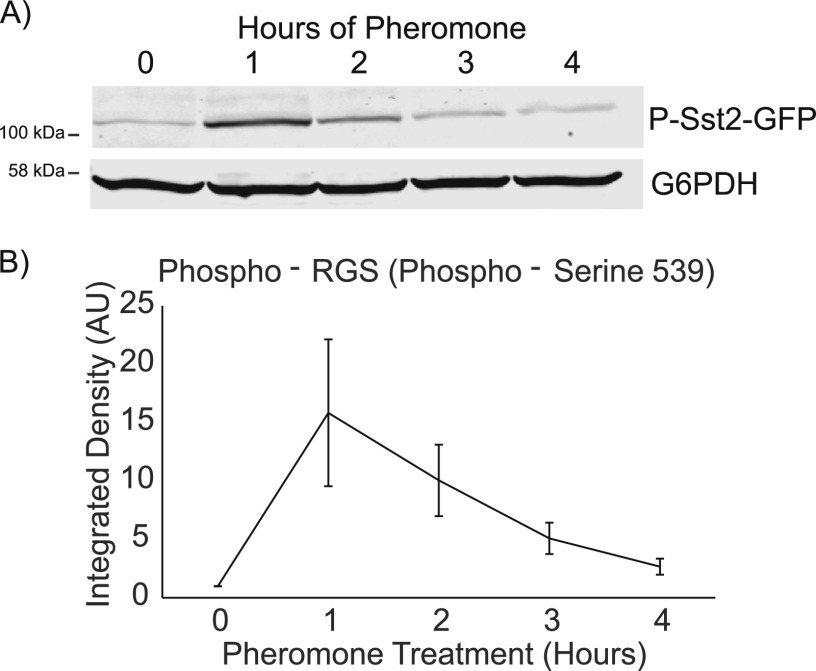
RGS phosphorylation peaks 1 h in the pheromone response. **(A)** Western blotting of phospho-RGS-GFP responding to saturating pheromone over 4 h. G6PDH was probed as a loading control. **(A, B)** Quantification of Western blotting shown in (A), normalized to G6PDH levels. Error bars represent standard error of the mean, n = 3.

To determine the dynamics of RGS phosphorylation, we treated an *SST2-GFP* strain with pheromone and took samples every hour for 4 h. We found that phosphorylation of the RGS peaks between 1 and 2 h, consistent with the literature (Garrison et al, 1999), but phosphorylation levels decrease to lower levels by 4 h post–pheromone treatment (Fig 6A and B). A complication of examining a decrease in pheromone-driven signaling is the desensitizing role of the protease Bar1, which degrades pheromone (Ciejek & Thorner, 1979). However, we found that this decrease was independent of Bar1 activity (Fig S3). Decreasing levels of phospho-RGS suggest that the role of the phosphorylated form of the RGS may be more important earlier in the pheromone response.

### Phosphorylation of RGS promotes coordination of cytokinesis with the pheromone response

**Figure 7.**
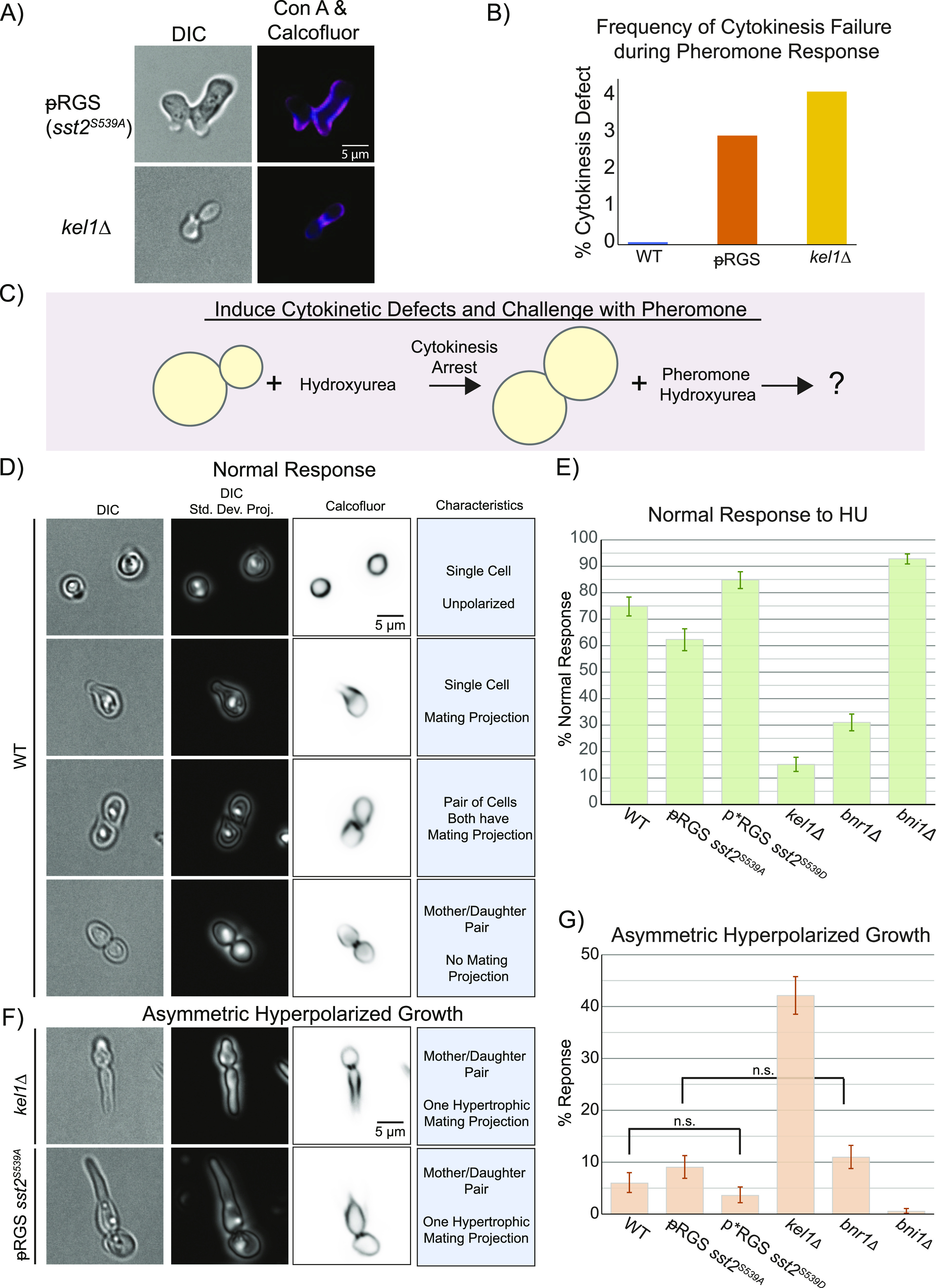
Phosphorylated Sst2 and the kelch-repeat protein Kel1 promote completion of cytokinesis before pheromone-induced polarization. **(A)** Images of ᵽRGS mutant and *kel1Δ* which have failed to complete cytokinesis before pheromone induce polarized growth. Cell walls were stained with Calcofluor white and concanavalin-A 647 to verify the open bud neck. **(B)** Wild-type and mutant ᵽRGS and *kel1Δ* strains were exposed to pheromone in culture for 90 min, fixed, and then failed cytokinetic events were counted. n = 1,412 (WT), 1,350 (ᵽRGS), and 1,396 (*kel1Δ*) from two separate experiments. **(C)** To drive cytokinetic defects, we pretreated cells with 100 mM hydroxyurea, followed by treatment with both hydroxyurea and pheromone to investigate the role of the indicated proteins in delay of pheromone-induced polarity until completed mitosis. **(D)** Images of normal phenotypes in response to HU + pheromone. Shown are a single focal plane of DIC, a SD projection of a stack of DIC images to better show the state of the bud neck, and cell wall staining with Calcofluor white. We considered a normal response to hydroxyurea and pheromone to be one of the following: (i) completion of cytokinesis but arrest as a circular cell, in the event that stress signaling is suppressing the pheromone response (a minority of cells). (ii) A lone cell responding to pheromone. (iii) Completion of cytokinesis (if the cells had resolved their DNA damage), followed by pheromone-induced morphogenesis. These cells may be still associated but show signs of completed cytokinesis. (iv) Arrest of cytokinesis yielding a mother daughter pair with no polarized growth. **(E)** Plots of the frequency of normal response to hydroxyurea and pheromone in the indicated strains. Error bars represent bootstrapped 95% confidence intervals. For each strain, n > 640 cells across three experiments. All differences are statistically significant for *P* < 0.05, as indicated by nonoverlapping 95% confidence intervals. **(F)** Examples of the asymmetric hyperpolarized growth phenotype. **(G)** Plots of the frequency of asymmetric hyperpolarized growth in response to hydroxyurea and pheromone. For samples with overlapping confidence intervals, statistical significance was tested by bootstrapping the 95% confidence interval of the difference in means. By this metric, we are 95% confident that WT and ᵽRGS (*sst2*^*S539A*^) have a nonzero difference in means (*P* < 0.05). Comparisons that are not statistically significant are marked “n.s.”

Cells which have already left G1 must complete mitosis and cytokinesis before polarizing and forming a mating projection in response to the pheromone. We found that some mother–daughter pairs in our unphosphorylatable ᵽRGS mutant (*sst2*^*S539A*^) formed mating projections before they had finished cytokinesis (Fig 7A). The frequency of the event was low, and we never observed these defects in wild-type cells or cells expressing the phospho-mimetic mutant during microfluidics experiments. Previous studies have found genetic interactions between the RGS and two proteins involved in cytokinesis, Bnr1 and Kel1 (Burchett et al, 2002; Yu et al, 2008). Both of these proteins play a role in mitosis, Bnr1 through the regulation of actin polymerization at the mitotic septin ring and Kel1 through promoting the MEN (Seshan et al, 2002; Pruyne et al, 2004; Buttery et al, 2007; Gao et al, 2010; Hotz & Barral, 2014). In addition, Kel1 serves as a negative regulator of Bnr1 and may impact cytokinesis through that role as well (Gould et al, 2014). Therefore, we hypothesized that the cytokinetic defect may be mediated through interactions with these proteins.

We examined *bnr1Δ* and *kel1Δ* cells responding to saturating pheromone and found that *kel1Δ* cells also occasionally fail to complete cytokinesis before responding to the pheromone (Fig 7A, negative data for *bnr1Δ* not shown). These were both rare events, and our microfluidic experiments do not contain large numbers of yeast, so we grew wild-type, ᵽRGS, and *kel1Δ* yeast in culture, treated with saturating pheromone, and counted the frequency of cells with failed cytokinesis based on visual inspection for conjoined yeast responding to the pheromone by DIC imaging. We found that both mutants lead to rates of failed cytokinesis of ∼3–4% (Fig 7B, minimum of 1,350 cells per strain). From this, we conclude that both Sst2 and Kel1 are both involved in a mechanism that ensures cytokinesis finishes before the pheromone response.

The rate of spontaneous cytokinetic defects under ideal growth conditions is relatively low. Thus, we took advantage of hydroxyurea (HU) that damages DNA and causes stalled cytokinesis (Amaral et al, 2016), forcing cells to contend with the competing signals of receptor-mediated polarity and an unresolved cytokinetic furrow. To test the role of phosphorylation of the RGS in promoting cytokinesis, we pretreated with 100 mM HU for 2 h followed by treatment with the pheromone (10 μM) while maintaining HU (Fig 7C). We then examined cells after 4 h of pheromone treatment to assess polarized growth and cytokinesis. We found that WT cells frequently stalled cytokinesis with two round cells joined at the bud neck. We scored phenotypes as a normal response if the cells showed evidence of completing cytokinesis before undergoing polarized growth, if they arrested as a mother–daughter pair with no evidence of polarized growth or if they had completed cytokinesis and began mating projection formation (Fig 7D and E). In cells with the unphosphorylatable ᵽRGS, we found more cells that had both failed cytokinesis and began polarized growth in one or both the mother and daughter cells. A particularly striking phenotype involves one cell remaining round, whereas the other shows hyperpolarized growth, which we refer to as asymmetric hyperpolarized growth (Fig 7F and G). The asymmetric hyperpolarized growth was suppressed by the phospho-mimetic p*RGS mutant (sst2^S539D^). Data sets with nonoverlapping 95% confidence intervals are statistically significant for *P* = 0.05 (Fig 7G). Both phospho-mutants have overlapping confidence intervals with the WT, which does not preclude a statistically significant difference. To compare these, we bootstrapped the confidence interval of the difference in means between each phospho-mutant strain and the wild-type strain. We then checked whether the 0 mean difference fell within the 95% confidence interval. If a difference of 0 falls outside of the 95% confidence interval, we rejected the null hypothesis and determined that the difference is statistically significant with a cutoff of *P* = 0.05. The differences in hyperpolarized growth between WT and the phospho-mimetic p*RGS were not statistically significant, but the increase in hyperpolarized growth in the unphosphorylatable ᵽRGS mutant compared with WT was statistically significant.

When we examined *kel1Δ* cells under these conditions, we found that the asymmetric hyperpolarized growth was a dominant phenotype (Fig 7G). Thus, unphosphorylatable ᵽRGS partially phenocopies the loss of Kel1 function. This suggests that phosphorylation of RGS may promote a Kel1-dependent mechanism that prevents the mating pathway from commandeering the polarity machinery before the completion of cytokinesis.

Kel1 has been identified as a negative regulator of the formin Bnr1 (Gould et al, 2014). Yeast have two formins: Bni1 is associated with the polar cap and is activated by Cdc42 and the Gα/MAPK complex (Breitsprecher & Goode, 2013). Bnr1 is associated with mitotic septin structures and has no known role in the pheromone pathway. Given the central role that formins play in both mitosis and the pheromone response, we hypothesized that the formins may facilitate the coordination of cytokinesis and the beginning of pheromone-induced polarized growth. We performed the same experiment as above, inducing cytokinetic defects with hydroxyurea followed by pheromone treatment, and assessed the ability of cells lacking either Bni1 or Bnr1 to prevent pheromone-induced polarization before the completion of cytokinesis. Deletion of Bni1 largely stopped polarization of cells before completion of cytokinesis and completely abrogated the asymmetric hyper-elongated phenotype (Fig 7E and G). Deletion of Bnr1 resulted in increased polarization before the completion of cytokinesis and increased levels of asymmetric hyperpolarized growth (Fig 7E and G). The asymmetric hyper-elongated growth phenotype is clearly dependent upon Bni1 and inhibited by Bnr1. Thus, coordination of pheromone-induced polarity with the completion of cytokinesis is promoted by Bnr1 function and antagonized by Bni1.

### The RGS Sst2 forms a complex with Kel1 that is enhanced by the unphosphorylatable S539A mutant

**Figure 8.**
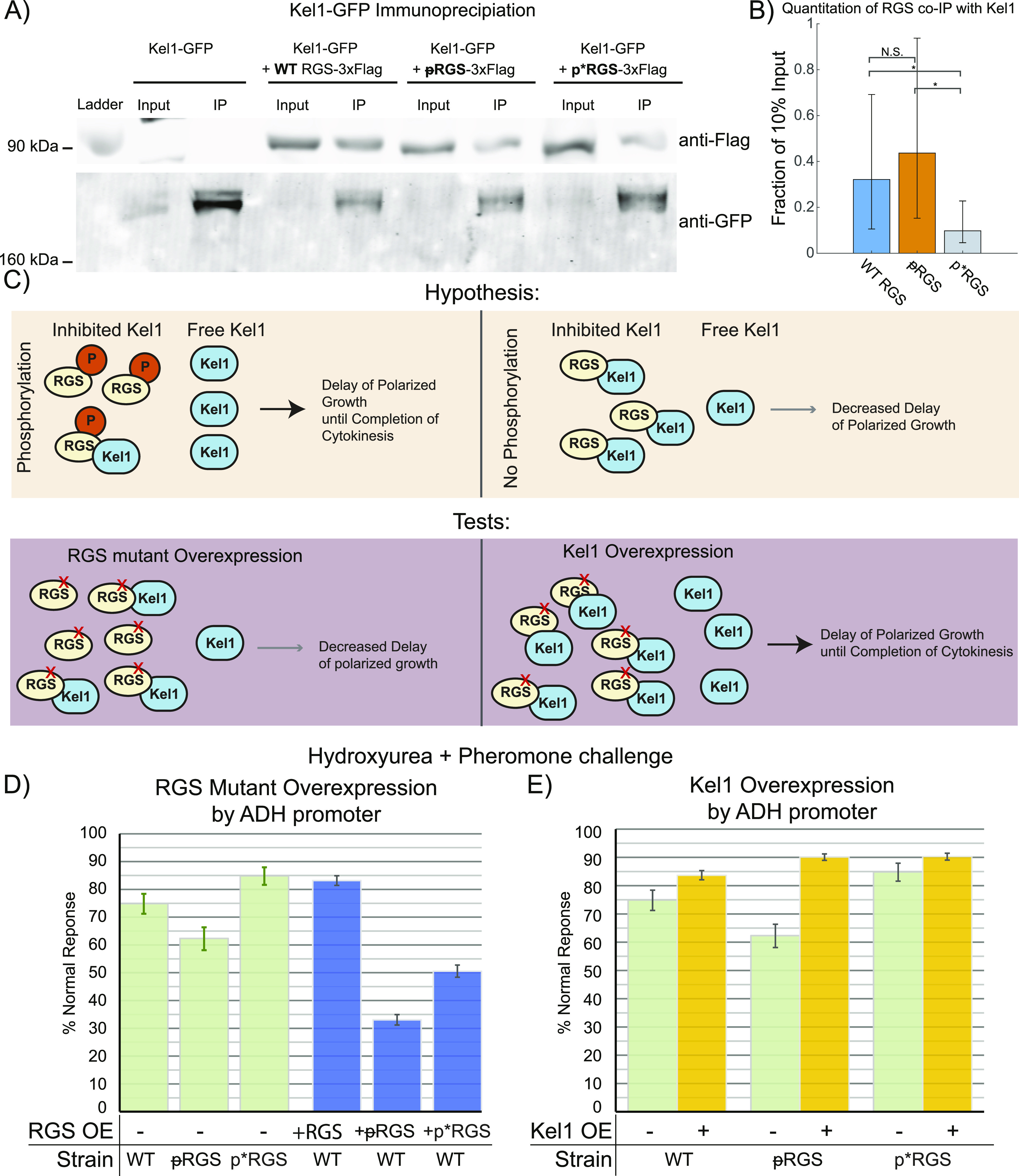
RGS and Kel1 form a complex and are in the same genetic pathway for regulation of cytokinesis. **(A)** Cells expressing Kel1-GFP and overexpressing RGS (pRSII416 pADH Sst2-3xFLAG) as either WT, ᵽRGS, or p*RGS were treated with pheromone for 1 h, lysed, and an immunoprecipitation was performed with a GFP antibody resin (GFP-trap). Westerns blots were probed for FLAG-RGS and for Kel1-GFP. Input lanes contain lysate equivalent to 10% of protein used for the immunoprecipitation. **(B)** Quantitation of the immunoprecipitation results from eight separate experiments. Error bars represent 95% confidence intervals. Because these confidence intervals overlap, statistical significance was tested by bootstrapping the 95% confidence interval of the difference in means. **(C)** Our data thus far lead us to the hypothesis that unphosphorylated RGS binds Kel1, inhibiting it, and that phosphorylated RGS binds less well, allowing more Kel1 to function for cytokinesis. This can be tested by overexpressing the unphosphorylatable ᵽRGS, which we would expect to decrease the delay in polarized growth and lead to increased cytokinetic defects. This hypothesis can also be tested by attempting to rescue cytokinetic defects in the ᵽRGS mutant by overexpressing Kel1. **(D)** We performed hydroxyurea and pheromone experiments as in Fig 7, with overexpression of WT and mutant RGS constructs from pRSII416 pADH RGS-3xFlag, as above. Graphed is the percentage of cells that carried out a normal response as previously defined. The green bars are data from Fig 7 for comparison. Error bars represent bootstrapped 95% confidence intervals. Data consist of n = 1784 cells for WT RGS, n = 2,398 for ᵽRGS, and n = 1,951 for p*RGS overexpression from three different experiments. **(D, E)** We carried out the same experiments in (D) but with Kel1 overexpressed in WT or mutant RGS backgrounds from pRSII416 pADH Kel1-3xFLAG. Green bars are data from Fig 7 for comparison. Error bars represent bootstrapped 95% confidence intervals. Data consist of n = 1,936 cells for WT RGS, n = 2,251 for ᵽRGS and n = 2,574 for p*RGS from three different experiments.

The above cytokinesis experiments show that Kel1 is important for the completion of cytokinesis before pheromone-induced polarity. The unphosphorylatable ᵽRGS (sst2^S539A^) shows a dominant negative effect on cytokinesis but with far less penetrance than Kel1 deletion. Although Kel1 has been found to interact genetically with Sst2 through yeast two-hybrid assays (Burchett et al, 2002; Yu et al, 2008), this has not been examined biochemically. To test whether the RGS forms a complex with Kel1, we generated cells expressing Kel1-GFP from the KEL1 genomic locus and a 3× FLAG–tagged RGS from the ADH1 promoter from a plasmid (pRSII416). The expressed RGS was WT RGS (Sst2-3XFLAG), ᵽRGS (sst2^S539A^-3XFLAG), or p*RGS (sst2^S539D^-3xFLAG). Cells were treated with pheromone for 1 h and lysed using a bead homogenizer. Kel1-GFP was immunoprecipitated using GFP-trap M270 magnetic resin. We performed SDS–PAGE and Western blotting on the immunoprecipitated sample and probed for the RGS with anti-FLAG antibody. We found that all forms of the RGS co-immunoprecipitated with Kel1 (Fig 8A), but we consistently recovered less of the phospho-mimetic mutant (Fig 8B).

Together, these data suggest the following hypothesis: Free Kel1 promotes proper cytokinesis, and unphosphorylated RGS binds to Kel1, inhibiting its function, whereas phosphorylation of the RGS relieves its inhibition of Kel1 (Fig 8C). This hypothesis would suggest that higher levels of the ᵽRGS would be more detrimental to cytokinesis, whereas higher levels of Kel1 would be predicted to rescue the defects induced by the unphosphorylatable ᵽRGS (Fig 8C).

We tested the overexpression of WT and phospho-mutant RGS using the ADH1 promoter-driven Sst2-3xFLAG plasmids described above in the HU/pheromone experiment as in Fig 7. Overexpression of WT RGS had minimal effect on the frequency of normal cytokinesis, whereas overexpression of either RGS phospho-mutant lead to much lower normal response to HU followed by pheromone. The unphosphorylatable ᵽRGS mutant resulted in the strongest disruption of normal response, whereas the phospho-mimetic p*RGS mutant lead to a larger defect than anticipated based on its behavior in other experiments (Fig 8D). It does still bind to Kel1 in the Co-IP experiments (Fig 8A and B), and so the overexpression may be sufficient to still have the dominant negative effect, or this may be a limitation of the aspartic acid mutation. In either case, the disruption of cytokinesis is clearly dependent upon the dose of RGS mutant present in the cell.

We tested the ability of Kel1 overexpression to rescue the RGS mutant defects in the HU/pheromone experiment by creating an ADH1 promoter-driven Kel1-3xFLAG plasmid. We transformed this plasmid into WT, ᵽRGS, and p*RGS strains and performed the HU and pheromone experiment as above. Kel1 overexpression was able to improve the response in every background (Fig 8E). Thus, Kel1 is genetically in the same pathway as the unphosphorylatable ᵽRGS for control of cytokinesis in the presence of pheromone.

## Discussion

Here, we set out to determine the role of feedback phosphorylation of the RGS Sst2. We found that the phosphorylation is dynamic through the pheromone response, reaching a maximal level between 1 and 2 h into the response (Fig 6). We found that phosphorylation of the RGS alters the localization of the RGS relative to the polar cap during the pheromone response and leads to a broadened distribution of the Gα-interacting MAPK Fus3 (Figs 4 and 5). Strikingly, cells unable to phosphorylate the RGS sometimes began polarized growth in response to pheromone without waiting for the completion of cytokinesis. By inducing cytokinetic defects with hydroxyurea, we were able to determine that phosphorylation of the RGS at serine 539 enhances the ability of the cell to stall cytokinesis without initiating pheromone induce polarity, thereby correctly integrating both an internal stress response and an external morphogenesis response (Fig 7). This coordination appears to use the kelch-repeat protein Kel1 and the formin Bnr1, whereas the formin Bni1 antagonizes completion of cytokinesis in the presence of pheromone. We found that the RGS forms a complex with Kel1 that is affected by serine 539 mutants and that cytokinetic defects are exacerbated by higher levels of RGS mutants, whereas higher levels of Kel1 can rescue unphosphorylatable ᵽRGS-induced cytokinetic defects (Fig 8).

### Coordination of the end of cytokinesis with the beginning of receptor-mediated morphogenesis

In an unsynchronized population, cells will be evenly distributed through the 90-min yeast cell cycle. Upon stimulation with the pheromone, receptor signaling will immediately begin with subsequent MAPK activation and downstream phosphorylation of the protein Far1 (Arkowitz, 2009). Far1 serves two purposes in the pheromone response: (1) to inhibit cyclin-dependent kinase activity, leading to arrest in G1 (Pope et al, 2014), and (2) to couple the Cdc42 GEF, Cdc24, to free Gβ, thereby promoting polarization to sites of active receptor (Nern & Arkowitz, 1999). The duration of receptor signaling before the repurposing of the polarity machinery will vary depending on where in the cell cycle each cell is when pheromone signaling begins. Thus, some cells may be an hour or more into pheromone signaling before completing cytokinesis, whereas others may be able to immediately start mating projection formation or experience a delay of only a few minutes. A potentially significant difference between these two scenarios is the amount of RGS present in the cell (Fig 2) as SST2 transcription is up-regulated by pheromone signaling (Dohlman et al, 1996), and so cells that must delay receptor-driven polarity for a long time before cytokinesis may be more prone to RGS-induced errors and be more dependent upon MAPK phosphorylation of the RGS. Indeed, our overexpression experiments suggest this is the case as higher levels of unphosphorylatable ᵽRGS leads to an increase in failed cytokinesis (Fig 8).

An obvious question arises from these findings: Does the RGS play a role in cytokinesis in the absence of pheromone? There are multiple lines of evidence to suggest that RGS has no role in normal cytokinesis. First, in previous studies on cells lacking the RGS, we have not observed any cytokinetic defects (Kelley et al, 2015). Second, baseline Sst2 levels are an order of magnitude higher in haploids than in diploids (de Godoy et al, 2008). If the RGS played a role in cytokinesis in the absence of pheromone, then haploid and diploid cells would need different mechanisms for regulating cytokinesis, an unlikely scenario.

Our data are consistent with unphosphorylated RGS inhibiting a subset of Kel1 function, as the unphosphorylatable ᵽRGS phenocopies the spontaneous failure to complete cytokinesis before mating projection formation that we see in cells lacking Kel1, and overexpression of this mutant enhances the penetrance of the phenotype (Figs 7 and 8). We would expect this inhibition of Kel1 to involve stochiometric binding (directly or through an intermediary), and so in the absence of pheromone, where RGS levels are low (Fig 2D and Dohlman et al, 1996), there would be very little impact of unphosphorylated RGS on Kel1 activity. Phosphorylation of the RGS, however, would prevent its inhibition of Kel1. We propose that RGS is phosphorylated early in the response to allow normal Kel1 function during the completion of the cell cycle.

Kel1 associates with the polar cap, regulates the formin Bnr1, is required for efficient mating, and takes part in the MEN (Philips & Herskowitz, 1998; Hofken & Schiebel, 2002; Gould et al, 2014; Smith & Rose, 2016). Kel1 contributes to the MEN by anchoring the Ras regulator Lte1 to the daughter cell during mitosis (Geymonat et al, 2010; Hotz & Barral, 2014). In addition to promoting mitotic exit, Kel1 and Lte1 have been found to suppress spurious polarization before the completion of mitosis, a role that may be separate from their role in MEN (Geymonat et al, 2010). Failure of Lte1 suppression of polarized growth leads to asymmetric hyperpolarized growth very similar to what we see in HU (Fig 7) (Geymonat et al, 2010). Future studies will be needed to examine whether LTE1 is responsible for the delay in receptor-mediated polarization to allow completion of cytokinesis.

The cytokinetic target of Kel1 may be the formin Bnr1 but Bnr1 appears to have little role in the pheromone pathway. Bni1 would seem a more likely candidate, and although there is no evidence that Kel1 can regulate Bni1, neither is there clear evidence that it cannot (Gould et al, 2014). Our hydroxyurea experiments show that the asymmetric hyperpolarized growth requires Bni1 and is inhibited by Kel1, consistent with a role for Kel1 negatively regulating Bni1 during the pheromone response (Fig 7).

### The role of RGS phosphorylation in the pheromone response

At later time points, we observed less phosphorylated RGS (Fig 6), suggesting a switch in the requirements for RGS as the pheromone response progresses. Our data suggest that the functional consequence of RGS phosphorylation altered spatial regulation of the pathway. Unphosphorylated RGS (e.g., WT at later time points or the ᵽRGS mutant) drives a larger distance between the MAPK Fus3 and the polar-cap marker Bem1, proteins that interact with active large G-protein (Gpa1) and active small G-protein (Cdc42), respectively (Fig 5). The simplest explanation of this observation is that the presence of RGS at the polar cap locally suppresses Gα activation. This is bolstered by the concentration of minimum MAPK concentration immediately proximal to the center of the polar cap, a phenomenon that is disrupted by the phospho-mimetic mutation that decreases RGS association with the polar cap. This type of negative feedback to the center of active signaling could help drive wandering of the polar cap by promoting large G-protein signaling further from the current site of polarization (Howell et al, 2012). Wandering of the polar cap is important for sensitive gradient tracking (Dyer et al, 2013), and so the small difference in the ability to track the gradient very well that we see in the p*RGS mutant (Fig 1) may be because of its decreased offset between the receptor-driven large G-protein and the Cdc42 driven polarity machinery. In addition, an offset between receptor signaling and the polar cap has recently been proposed to play a role in gradient tracking (Ghose et al, 2021), and although we did not see an effect on gradient tracking here, under more difficult tracking conditions, the observed Gα offset may enhance chemotropic growth.

Our data are consistent with unphosphorylated RGS inhibiting Kel1 function later in the pheromone response. Recent work by Garcia et al (2021) has found that Kel1 suppresses spontaneous activation of the pheromone pathway and suppresses noise in the pheromone pathway, both roles that have been previously identified for the RGS Sst2 (Chan & Otte, 1982; Dixit et al, 2014). Given our finding that Kel1 and Sst2 form a complex, it may be that Kel1 promotes Sst2 function more broadly.

In conclusion, here, we have established a role for the phosphorylation of the RGS Sst2 at serine 539 in promoting the completion of cytokinesis before the pheromone-induced pathway, repurposing the mitotic Cdc42 machinery for production of the mating projection or shmoo. When we exacerbate the cytokinetic defect using a DNA-damaging agent, the cell must integrate competing signals: (1) a checkpoint instructing the cell to stop mitosis and (2) a GPCR signaling pathway instructing the cell to polarize toward a mating partner. After completion of cytokinesis, the yeast polarize and respond to pheromone, where the interaction between Kel1 and RGS alters the spatial signaling of the MAPK Fus3.

Table S1 Yeast strains used in this study.

## Materials and Methods

### Yeast strains

Strains used in this study are shown in Table S1. Strains were constructed in the MATa haploid *S. cerevisiae* strain BY4741 using the plasmids in Table S2. Proteins were tagged with EGFP or mRuby2 at the native chromosomal locus through oligonucleotide-directed homologous recombination with using the primers listed in Table S3. For tagging Bem1 with Ruby, we created the integrating plasmid pRSII-Bem1-yomRuby2-Kan (Table S2). Bem1 nucleic acids 522–1,653 were cloned into pRSII405 (Chee & Haase, 2012) followed by link-yomRuby2 from pFA6a-link-yomRuby2 (Lee et al, 2013) using the primers indicated in Table S3. GFP tagging was generated by using pFA6a-link-yoEGFP-spHis5 Kan (Lee et al, 2013) or by amplifying the GFP cassette from the yeast GFP collection (Huh et al, 2003). pFA6a-link-yomRuby2-Kan was a gift from Wendell Lim & Kurt Thorn (Addgene plasmid # 44953; http://n2t.net/addgene:44953; RRID:Addgene_44953). pRSII405 was a gift from Steven Haase (Addgene plasmid # 35440; http://n2t.net/addgene:35440; RRID:Addgene_35440).

Table S2 Plasmids used in this study.

Table S3 Oligonucleotides used in this study.

Sst2 phospho-mutants were made by integrating the codon of interest with a PCR-amplified CORE cassette (Storici & Resnick, 2006). Deletions were performed by first amplifying the genomic locus from the MATa haploid deletion collection (Dharmacon) with primers listed in Table S3 and transformed using a standard lithium acetate transformation (Burke et al, 2005).

Cells were grown in rich medium (YPD) or synthetic medium (SC) at 30°C unless otherwise indicated. PCR products were transformed into yeast strains using standard lithium acetate transformation procedure. Individual colonies were isolated by growth on standard selective media (SC leu-, SC ura-, SC his-), selective media with 5-fluoroorotic acid (Zymo Research), or YPD selective media (YPD G418+). Transformants were verified using fluorescence microscopy, sequencing, and/or PCR.

### Hydroxyurea experiments

Yeast cultures were grown to an OD600 of 0.4–0.6 at 30°C and then pretreated with 100 mM hydroxyurea (Alfa Aesar) for 2 h at 30°C. After 2 h of pre-treatment with hydroxyurea (HU), a saturating concentration of α-factor (10 μM) was added, and cultures were then fixed after 240 min using an overnight ethanol fixation at −20°C. After ethanol fixation, yeast were resuspended and washed twice in 50 mM sodium citrate buffer (pH 7.2). Next, the cultures were incubated with 20 mg/ml RNase A (Thermo Fisher Scientific) for a minimum of 1 h at 37°C. After RNase incubation, proteinase K (Thermo Fisher Scientific) was added to the cultures at a final concentration of 0.4 mg/ml and incubated at 55°C for a minimum of 1 h then placed at 4°C overnight. For imaging, cells were pelleted, then washed and mounted in 1× PBS (pH 7.4). Cells were then imaged on the IX83 epifluorescence microscope (Olympus).

### Spontaneous cytokinesis defect experiments

Yeast strains were grown in liquid synthetic complete media with 2% dextrose (SCD) at 30°C to an OD600 of 0.6–0.8. Cells were treated with 30 μM α-factor for 90 min. Cells then were fixed in 4% paraformaldehyde, 2% glucose, and 30 μM α-factor for 20 min. After fixation and three washes with 1× PBS, the cells were stained with 7 μM Calcofluor white for 20 min and 50 μg/ml of concanavalin-A (both obtained from Biotium) for 30 min. The cells were once again washed three times with 1× PBS and then imaged. Randomly chosen fields were imaged, and then cells were scored for failed cytokinesis.

### Antibody production

The following peptides corresponding to the Sst2 amino acid sequence surround serine 539 were synthesized by Genscript, the phospho-Sst2 S539 peptide LHPH**S**PLSEC, where the **S** was phosphorylated, and the unphosphorylated peptide LHPHSPLSEC. The phospho-peptide was injected into rabbits by Cocalico Biologicals according to their standard protocol. The antibody was affinity purified on phospho-peptide covalently bound to a SulfoLink column according to the manufacturer’s instructions (Thermo Fisher Scientific).

### Western blotting

The phosphorylation state of Sst2 was assessed by Western blotting. Yeast cultures were grown overnight in 30°C. Cells were lysed with TCA buffer, and protein concentrations were determined using the DC protein assay kit (Bio-Rad). Protein separation was performed with a 7.5% SDS–PAGE and transferred to nitrocellulose at 100 V for 90 min. The primary antibody (1:1,000) and non-phospho-peptide (1:10,000) were incubated in 1% PBST blocking solution overnight followed by secondary-antibody incubation (1:10,000) in 1% PBST blocking solution for 1 h. Band intensity was detected via Odyssey CLx imaging system (LI-COR) and then quantified using ImageJ.

### Imaging on agarose pad

Yeast were imaged on an Olympus IX83 with a 60X-TIRF 1.49 NA objective, a Photometrics Prime 95b camera, X-Cite LED 120 Boost fluorescence light source (Excelitas), and filters for DAPI and GFP (Semrock). Cells were grown to mid-log phase (OD600 = 0.1–0.8) at 30°C in Synthetic Complete Media with 2% dextrose (SCD) and then imaged on pads made of 2% agarose in SCD as the use of agarose leads to lower autofluorescence than standard agar pads. Imaging was performed with an objective heater (Bioptechs) set to 30°C. Cells were pelleted and then resuspended in SC with 3 μM α-factor and placed on an agarose pad as above.

### Microfluidics experiments

Microfluidic devices were made by using a silicone polymer poured onto a microfluidics device mold (Suzuki et al, 2021) fabricated by UMaine FIRST. SYLGARD 184 Silicone Polymer was mixed at a ratio of 10:1, part A to part B, using a glass stirring rod to mix (Dow). Mixed polymer was poured onto the device mold and placed in a vacuum chamber for 1 h. After all air bubbles were removed, the mixture was placed in an oven at 80°C for 1 h. After cooling to room temperature, devices were cut out using a razor, and ports were punctured using an 18-gauge luer stub. Prepped devices and coverslips were cleaned by spraying with methanol, ethanol, then water, and dried using an air hose. Devices and coverslips were exposed to oxygen plasma for 45 s in a Harrick Plasma PDC32G Cleaner followed by fusion of the device to the cover slip.

Cultures were grown in SC to an OD600 between 0.1–0.8 at 30°C. Live-cell microfluidics experiments were performed using an IX83 (Olympus) microscope with a Prime 95B CMOS Camera (Photometrics) controlled by Cell Sens 1.17 (Olympus). Fluorescence and differential interference contrast (DIC) images were acquired using an Olympus-APON-60X-TIRF objective. Z-stacks of GFP and RFP images were acquired using an X-Cite 120 LEDBoost (Excelitas). Cells were imaged in a microfluidic device based on the dial-a-wave design that allows for the rapid switching of media while holding the yeast in place (Bennett et al, 2008; Dixit et al, 2014; Suzuki et al, 2021). Pheromone addition was verified using AlexaFluor 647 dye (Life Technologies) imaged with a single plane image. Cells were imaged at 20-min intervals for 12 h for 300 nM experiments and 5-min intervals for 0–150 nM experiments. Confocal microscopy was conducted on a Leica DMi8 (Leica) imaging platform equipped with an automated stage, SP8X white-light laser (capped at 70% of total power), an argon laser (Leica Microsystems). All imaging was conducted using HyD hybrid detectors. Imaging settings were determined based on experimental needs and were replicated for repeat experiments.

### Image analysis

Images were deconvolved using the Huygens software (Scientific Volume Imaging) Classic Maximum Likelihood Estimation (CMLE) deconvolution algorithm. Masks of cells were made using ImageJ (Schindelin et al, 2012), and data analysis was performed using MATLAB (MathWorks). To quantify the fraction of protein localization over time, MATLAB was used as described in Figs S1 and S2 and previously (Kelley et al, 2015; Shellhammer et al, 2019). The fluorescent intensity of each fluorescent protein was extracted over time using a line width of 5 pixels. Peak Bem1 was used as a reference to normalize the spatial distribution of proteins of interest in relation to the polar cap. This was done by setting peak Bem1 as the midpoint and shifting the protein of interest in the same manner. For profiles reporting fraction of protein at each position, fluorescence was normalized by subtracting the minimum value from each line-scan, followed by normalization of the subtracted data to sum to one. The normalized fluorescence intensity was plotted at each point along the cell periphery with shaded regions showing 95% confidence intervals derived by bootstrapping with 10,000 resamplings. Statistical analysis was performed between profiles using a sliding one-way ANOVA and Tukey’s honestly significant difference test followed by false discovery rate adjustment with the MATLAB *mafdr()* function with *P*-values < 0.05 denoted as significant. Where indicated, a pairwise Kolmogorov–Smirnov test was performed using the MATLAB *kstest2()* function. When excluding nuclear fluorescence from Fus3-GFP images, we modified the “granulinator” script (Hunn et al, 2022) to select nuclei for removal. We used cell masks to calculate fluorescence histograms for each cell and adjusted the size (minimum size of 25 pixels) and threshold (1 SD above mean) cutoffs to detect nuclei but not polar caps. The resultant mask was enlarged by a pixel to ensure elimination of peripheral nuclear signal. The area masked was then replaced with the average fluorescence of the cell.

### Plasmid construction

The plasmids used for overexpression of Sst2 or Kel1 by the ADH1 promoter were constructed using the NEB Gibson Assembly Cloning Kit (E2611S; NEB) as advised by the manufacturer’s instructions. All plasmids were built using the pRSII416 vector backbone (Plasmid #: 35456; Addgene [Chee & Haase, 2012]). The vector backbone was linearized with SacI-HF (R3156S; NEB) and ApaI (R0114S; NEB) restriction enzymes before Gibson assembly. Primers were constructed using the online NEBuilder assembly tool (v2.6.0, https://nebuilder.neb.com/) and are listed in Table S3. The forward and reverse 3XFLAG sequences were obtained from p3xFLAG-CMV-14 and synthesized as oligos for PCR amplification with primers in Table S3. The 1 kilobase of DNA upstream of the ADH1 was amplified from genomic DNA to provide the ADH1 promoter.

### Co-immunoprecipitation

Cells expressing Kel1-GFP and transformed with pRSII416-based plasmids overexpressing Sst2-3XFLAG, Sst2^S539A^-3XFLAG, or Sst2^S539D^-3XFLAG (Table S2) were grown to an OD600 of 0.2–0.8 in synthetic complete +dextrose-leucine media at 30°C. Cells were then treated with 10 μM α-factor for 1 h of shaking at 30°C. Pheromone-treated cells were immediately placed on ice then centrifuged at max speed in an Eppendorf 5420R (Eppendorf) swinging-bucket centrifuge for 5 min at 4°C, and supernatant was subsequently aspirated. Cell pellets were resuspended with 1.5 times the pellet volume of lysis buffer (10 mM Tris pH 7.4, 150 mM NaCl, 0.5 mM EDTA) containing 1X HALT protease inhibitor (78429; Thermo Fisher Scientific) and 1X phosphatase inhibitor (J61022.AA; Alfa Aesar) and transferred to a reinforced screw-cap tube (15-340-162; Fisherbrand) containing 100 μl acid-washed glass beads (G8772; Sigma-Aldrich). Cells were homogenized using a Bead Mill 4 homogenizer (Thermo Fisher Scientific) at max speed for 3 × 40 s rounds with 60-s rest cycles on ice. Homogenization was conducted at 4°C. After homogenization, Triton X-100 was added to the whole-cell lysate to a final concentration of 0.5% and followed by a 30-min rotating incubation at 4°C. After incubation, whole-cell lysate was transferred to a 1.5-ml tube and centrifuged at max speed for 20 min at 4°C. The resultant supernatant was transferred to a final tube and kept on ice. Protein concentration was determined using a DC Protein Assay kit (Bio-Rad), and assay was conducted in triplicate using a Biotek Synergy 2 plate reader (Biotek).

After the protein assay, GFP-Trap Magnetic Particles M-270 (ChromoTek) were washed three times with IP Lysis Buffer containing protease and phosphatase inhibitors and raised in 100 μL IP Lysis Buffer containing 200 μg protein lysate. GFP-Trap particles were incubated for 1 h at 4°C on a rotator. GFP-trap particles were washed three-times with lysis buffer containing protease and phosphatase inhibitors, and then bound proteins were solubilized in 2× Laemmli sample buffer. Input samples containing whole-cell lysate were made with 20 μg total protein in 1× sample Laemmli. Samples were boiled for 5 min at 95°C, allowed to cool to room temperature, and spun at max speed for 1 min using an Eppendorf 5424 centrifuge (Eppendorf).

Protein separation was performed using 8% SurePAGE Bis-Tris gels (GenScript) and MOPS buffer (containing 5 mM sodium bisulfite) followed by transfer to low-fluorescence PVDF membranes (Immobilon FL; Millipore) using Towbin Transfer Buffer containing 0.1% SDS at 100 V for 150 min on ice. Membranes were incubated in Ponceau S (BP103-10; Fisher BioReagents) on a rocker for 5 min at room temperature then washed with a 10% acetic acid solution rocking for 10 min before imaging. Membranes were blocked with a 5% milk solution in PBS-T (1× PBS+0.1% Tween-20) for 1 h at room temperature. Membranes were then incubated overnight at 4°C in a 1% milk solution in PBS-T containing 1:1,000 Rabbit anti-GFP (2956S; Cell Signaling) and 1:1,000 Mouse anti-M2-FLAG (F1804; Sigma-Aldrich). After overnight primary antibody incubation, membranes were washed three times with PBS-T for 5 min each and incubated with 1% milk in PBS-T containing 1:5,000 Donkey anti-Rabbit 800 CW (926-32213; LI-COR) and 1:5,000 Donkey anti-Mouse 680 RD (926-68072; LI-COR) secondary antibodies for 1 h at room temperature. Finally, membranes were washed two times with PBS-T for 5 min and once with PBS for 5 min before imaging using at 42-μm resolution and “High” quality settings on a Li-COR Odyssey CLx imaging system (Li-COR Biosciences). Quantitation of integrated density was conducted using FIJI’s gel analysis tool. The fraction of GFP-Trap bound Sst2-3XFLAG was normalized to the input fraction. Data from eight replicate experiments were averaged, and error bars were constructed by bootstrapping the 95% confidence interval in MATLAB. Statistical significance was assessed by bootstrapping the 95% confidence of a nonzero difference in means.

## Data Availability

The MATLAB scripts necessary for data and image analysis in this work are available at https://github.com/Kelley-Lab-Computational-Biology/RGS_Phosphorylation.

## Supplementary Material

Reviewer comments

## References

[bib1] Alvaro CG, Thorner J (2016) Heterotrimeric G protein-coupled receptor signaling in yeast mating pheromone response. J Biol Chem 291: 7788–7795. 10.1074/jbc.r116.714980 26907689 PMC4824985

[bib2] Amaral N, Vendrell A, Funaya C, Idrissi FZ, Maier M, Kumar A, Neurohr G, Colomina N, Torres-Rosell J, Geli MI, (2016) The Aurora-B-dependent NoCut checkpoint prevents damage of anaphase bridges after DNA replication stress. Nat Cell Biol 18: 516–526. 10.1038/ncb3343 27111841

[bib3] Apanovitch DM, Slep KC, Sigler PB, Dohlman HG (1998) Sst2 is a GTPase-activating protein for Gpa1: Purification and characterization of a Cognate RGS−Gα protein pair in yeast. Biochemistry 37: 4815–4822. 10.1021/bi9729965 9537998

[bib4] Arkowitz RA (2009) Chemical gradients and chemotropism in yeast. Cold Spring Harbor Perspect Biol 1: a001958. 10.1101/cshperspect.a001958 PMC2742094 20066086

[bib5] Ballon DR, Flanary PL, Gladue DP, Konopka JB, Dohlman HG, Thorner J (2006) DEP-domain-mediated regulation of GPCR signaling responses. Cell 126: 1079–1093. 10.1016/j.cell.2006.07.030 16990133

[bib6] Bar-Shavit R, Maoz M, Kancharla A, Nag JK, Agranovich D, Grisaru-Granovsky S, Uziely B (2016) G protein-coupled receptors in cancer. Int J Mol Sci 17: 1320. 10.3390/ijms17081320 PMC5000717 27529230

[bib7] Bennett MR, Pang WL, Ostroff NA, Baumgartner BL, Nayak S, Tsimring LS, Hasty J (2008) Metabolic gene regulation in a dynamically changing environment. Nature 454: 1119–1122. 10.1038/nature07211 18668041 PMC2654342

[bib8] Breitsprecher D, Goode BL (2013) Formins at a glance. J Cell Sci 126: 1–7. 10.1242/jcs.107250 23516326 PMC3603506

[bib9] Burchett SA, Flanary P, Aston C, Jiang L, Young KH, Uetz P, Fields S, Dohlman HG (2002) Regulation of stress response signaling by the N-terminal dishevelled/EGL-10/pleckstrin domain of Sst2, a regulator of G protein signaling in Saccharomyces cerevisiae. J Biol Chem 277: 22156–22167. 10.1074/jbc.m202254200 11940600

[bib10] Burke D, Dawson D, Stearns T (2005) Methods in Yeast Genetics: A Cold Spring Harbor Laboratory Course Manual. Cold Spring Harbor, NY: Cold Spring Harbor Press.

[bib11] Buttery SM, Yoshida S, Pellman D (2007) Yeast formins Bni1 and Bnr1 utilize different modes of cortical interaction during the assembly of actin cables. Mol Biol Cell 18: 1826–1838. 10.1091/mbc.e06-09-0820 17344480 PMC1855024

[bib12] Chan RK, Otte CA (1982) Physiological characterization of Saccharomyces cerevisiae mutants supersensitive to G1 arrest by a factor and alpha factor pheromones. Mol Cell Biol 2: 21–29. 10.1128/mcb.2.1.21-29.1982 7050666 PMC369749

[bib13] Chasse SA, Flanary P, Parnell SC, Hao N, Cha JY, Siderovski DP, Dohlman HG (2006) Genome-scale analysis reveals Sst2 as the principal regulator of mating pheromone signaling in the yeast Saccharomyces cerevisiae. Eukaryot Cell 5: 330–346. 10.1128/ec.5.2.330-346.2006 16467474 PMC1405904

[bib14] Chee MK, Haase SB (2012) New and redesigned pRS plasmid shuttle vectors for genetic manipulation of saccharomycescerevisiae. G3 (Bethesda) 2: 515–526. 10.1534/g3.111.001917 22670222 PMC3362935

[bib15] Chiou JG, Balasubramanian MK, Lew DJ (2017) Cell polarity in yeast. Annu Rev Cell Dev Biol 33: 77–101. 10.1146/annurev-cellbio-100616-060856 28783960 PMC5944360

[bib16] Ciejek E, Thorner J (1979) Recovery of S. cerevisiae a cells from G1 arrest by alpha factor pheromone requires endopeptidase action. Cell 18: 623–635. 10.1016/0092-8674(79)90117-x 391400

[bib17] de Godoy LMF, Olsen JV, Cox J, Nielsen ML, Hubner NC, Frohlich F, Walther TC, Mann M (2008) Comprehensive mass-spectrometry-based proteome quantification of haploid versus diploid yeast. Nature 455: 1251–1254. 10.1038/nature07341 18820680

[bib18] DiBello PR, Garrison TR, Apanovitch DM, Hoffman G, Shuey DJ, Mason K, Cockett MI, Dohlman HG (1998) Selective uncoupling of RGS action by a single point mutation in the G protein alpha-subunit. J Biol Chem 273: 5780–5784. 10.1074/jbc.273.10.5780 9488712

[bib19] Dixit G, Kelley JB, Houser JR, Elston TC, Dohlman HG (2014) Cellular noise suppression by the regulator of G protein signaling Sst2. Mol Cell 55: 85–96. 10.1016/j.molcel.2014.05.019 24954905 PMC4142594

[bib20] Dohlman HG, Apaniesk D, Chen Y, Song J, Nusskern D (1995) Inhibition of G-protein signaling by dominant gain-of-function mutations in Sst2p, a pheromone desensitization factor in Saccharomyces cerevisiae. Mol Cell Biol 15: 3635–3643. 10.1128/mcb.15.7.3635 7791771 PMC230601

[bib21] Dohlman HG, Song J, Ma D, Courchesne WE, Thorner J (1996) Sst2, a negative regulator of pheromone signaling in the yeast Saccharomyces cerevisiae: Expression, localization, and genetic interaction and physical association with Gpa1 (the G-protein alpha subunit). Mol Cell Biol 16: 5194–5209. 10.1128/mcb.16.9.5194 8756677 PMC231520

[bib22] Dyer JM, Savage NS, Jin M, Zyla TR, Elston TC, Lew DJ (2013) Tracking shallow chemical gradients by actin-driven wandering of the polarization site. Curr Biol 23: 32–41. 10.1016/j.cub.2012.11.014 23200992 PMC3543483

[bib23] Elion EA, Satterberg B, Kranz JE (1993) FUS3 phosphorylates multiple components of the mating signal transduction cascade: Evidence for STE12 and FAR1. Mol Biol Cell 4: 495–510. 10.1091/mbc.4.5.495 8334305 PMC300953

[bib24] Errede B, Vered L, Ford E, Pena MI, Elston TC (2015) Pheromone-induced morphogenesis and gradient tracking are dependent on the MAPK Fus3 binding to Gα. Mol Biol Cell 26: 3343–3358. 10.1091/mbc.e15-03-0176 26179918 PMC4569322

[bib25] Gao L, Liu W, Bretscher A (2010) The yeast formin Bnr1p has two localization regions that show spatially and temporally distinct association with septin structures. Mol Biol Cell 21: 1253–1262. 10.1091/mbc.e09-10-0861 20147448 PMC2847528

[bib26] Garcia I, Orellana-Munoz S, Ramos-Alonso L, Andersen AN, Zimmermann C, Eriksson J, Boe SO, Kaferle P, Papamichos-Chronakis M, Chymkowitch P, (2021) Kel1 is a phosphorylation-regulated noise suppressor of the pheromone signaling pathway. Cell Rep 37: 110186. 10.1016/j.celrep.2021.110186 34965431

[bib27] Garrison TR, Zhang Y, Pausch M, Apanovitch D, Aebersold R, Dohlman HG (1999) Feedback phosphorylation of an RGS protein by MAP kinase in yeast. J Biol Chem 274: 36387–36391. 10.1074/jbc.274.51.36387 10593933

[bib28] Geymonat M, Spanos A, Jensen S, Sedgwick SG (2010) Phosphorylation of Lte1 by Cdk prevents polarized growth during mitotic arrest in S. cerevisiae. J Cell Biol 191: 1097–1112. 10.1083/jcb.201005070 21149565 PMC3002025

[bib29] Ghose D, Jacobs K, Ramirez S, Elston T, Lew D (2021) Chemotactic movement of a polarity site enables yeast cells to find their mates. Proc Natl Acad Sci U S A 118: e2025445118. 10.1073/pnas.2025445118 34050026 PMC8179161

[bib30] Gould CJ, Chesarone-Cataldo M, Alioto SL, Salin B, Sagot I, Goode BL (2014) Saccharomyces cerevisiae Kelch proteins and Bud14 protein form a stable 520-kDa formin regulatory complex that controls actin cable assembly and cell morphogenesis. J Biol Chem 289: 18290–18301. 10.1074/jbc.m114.548719 24828508 PMC4140247

[bib31] Hao N, Nayak S, Behar M, Shanks RH, Nagiec MJ, Errede B, Hasty J, Elston TC, Dohlman HG (2008) Regulation of cell signaling dynamics by the protein kinase-scaffold Ste5. Mol Cell 30: 649–656. 10.1016/j.molcel.2008.04.016 18538663 PMC2518723

[bib32] Hauser AS, Attwood MM, Rask-Andersen M, Schioth HB, Gloriam DE (2017) Trends in GPCR drug discovery: New agents, targets and indications. Nat Rev Drug Discov 16: 829–842. 10.1038/nrd.2017.178 29075003 PMC6882681

[bib33] Hofken T, Schiebel E (2002) A role for cell polarity proteins in mitotic exit. EMBO J 21: 4851–4862. 10.1093/emboj/cdf481 12234925 PMC126280

[bib34] Hotz M, Barral Y (2014) The mitotic exit network: New turns on old pathways. Trends Cell Biol 24: 145–152. 10.1016/j.tcb.2013.09.010 24594661

[bib35] Howell A, Jin M, Wu C-F, Zyla T, Elston T, Lew D (2012) Negative feedback enhances robustness in the yeast polarity establishment circuit. Cell 149: 322–333. 10.1016/j.cell.2012.03.012 22500799 PMC3680131

[bib36] Huh WK, Falvo JV, Gerke LC, Carroll AS, Howson RW, Weissman JS, O’Shea EK (2003) Global analysis of protein localization in budding yeast. Nature 425: 686–691. 10.1038/nature02026 14562095

[bib37] Hunn JC, Hutchinson KM, Kelley JB, Reines D (2022) Variable penetrance of Nab3 granule accumulation quantified by a new tool for high-throughput single-cell granule analysis. Curr Genet 68: 467–480. 10.1007/s00294-022-01234-2 35301575 PMC9283369

[bib38] Kelley JB, Dixit G, Sheetz JB, Venkatapurapu SP, Elston TC, Dohlman HG (2015) RGS proteins and septins cooperate to promote chemotropism by regulating polar cap mobility. Curr Biol 25: 275–285. 10.1016/j.cub.2014.11.047 25601550 PMC4318785

[bib39] Lappano R, Maggiolini M (2012) GPCRs and cancer. Acta Pharmacol Sin 33: 351–362. 10.1038/aps.2011.183 22266725 PMC4077134

[bib40] Lee S, Lim WA, Thorn KS (2013) Improved blue, green, and red fluorescent protein tagging vectors for S. cerevisiae. PLoS One 8: e67902. 10.1371/journal.pone.0067902 23844123 PMC3699464

[bib41] Matheos D, Metodiev M, Muller E, Stone D, Rose MD (2004) Pheromone-induced polarization is dependent on the Fus3p MAPK acting through the formin Bni1p. J Cell Biol 165: 99–109. 10.1083/jcb.200309089 15067022 PMC2172092

[bib42] Metodiev MV, Matheos D, Rose MD, Stone DE (2002) Regulation of MAPK function by direct interaction with the mating-specific Gα in yeast. Science 296: 1483–1486. 10.1126/science.1070540 12029138

[bib43] Nern A, Arkowitz RA (1999) A Cdc24p-Far1p-Gβγ protein complex required for yeast orientation during mating. J Cell Biol 144: 1187–1202. 10.1083/jcb.144.6.1187 10087263 PMC2150586

[bib44] Park HO, Bi E (2007) Central roles of small GTPases in the development of cell polarity in yeast and beyond. Microbiol Mol Biol Rev 71: 48–96. 10.1128/mmbr.00028-06 17347519 PMC1847380

[bib45] Parnell SC, Marotti LA, Kiang L, Torres MP, Borchers CH, Dohlman HG (2005) Phosphorylation of the RGS protein Sst2 by the MAP kinase Fus3 and use of Sst2 as a model to analyze determinants of substrate sequence specificity. Biochemistry 44: 8159–8166. 10.1021/bi0503091 15924435

[bib46] Peter M, Gartner A, Horecka J, Ammerer G, Herskowitz I (1993) FAR1 links the signal transduction pathway to the cell cycle machinery in yeast. Cell 73: 747–760. 10.1016/0092-8674(93)90254-n 8500168

[bib47] Philips J, Herskowitz I (1998) Identification of Kel1p, a kelch domain-containing protein involved in cell fusion and morphology in Saccharomyces cerevisiae. J Cell Biol 143: 375–389. 10.1083/jcb.143.2.375 9786949 PMC2132843

[bib48] Pope PA, Bhaduri S, Pryciak PM (2014) Regulation of cyclin-substrate docking by a G1 arrest signaling pathway and the Cdk inhibitor Far1. Curr Biol 24: 1390–1396. 10.1016/j.cub.2014.05.002 24909323 PMC4086830

[bib49] Pruyne D, Gao L, Bi E, Bretscher A (2004) Stable and dynamic axes of polarity use distinct formin isoforms in budding yeast. Mol Biol Cell 15: 4971–4989. 10.1091/mbc.e04-04-0296 15371545 PMC524755

[bib50] Schindelin J, Arganda-Carreras I, Frise E, Kaynig V, Longair M, Pietzsch T, Preibisch S, Rueden C, Saalfeld S, Schmid B, (2012) Fiji: An open-source platform for biological-image analysis. Nat Methods 9: 676–682. 10.1038/nmeth.2019 22743772 PMC3855844

[bib51] Segall JE (1993) Polarization of yeast cells in spatial gradients of alpha mating factor. Proc Natl Acad Sci U S A 90: 8332–8336. 10.1073/pnas.90.18.8332 8397402 PMC47350

[bib52] Seshan A, Bardin AJ, Amon A (2002) Control of Lte1 localization by cell polarity determinants and Cdc14. Curr Biol 12: 2098–2110. 10.1016/s0960-9822(02)01388-x 12498684

[bib53] Shellhammer JP, Pomeroy AE, Li Y, Dujmusic L, Elston TC, Hao N, Dohlman HG (2019) Quantitative analysis of the yeast pheromone pathway. Yeast 36: 495–518. 10.1002/yea.3395 31022772 PMC6684483

[bib54] Smith JA, Rose MD (2016) Kel1p mediates yeast cell fusion through a Fus2p- and Cdc42p-dependent mechanism. Genetics 202: 1421–1435. 10.1534/genetics.115.185207 26865368 PMC4905532

[bib55] Storici F, Resnick MA (2006) The delitto perfetto approach to in vivo site-directed mutagenesis and chromosome rearrangements with synthetic oligonucleotides in yeast. Methods Enzymol 409: 329–345. 10.1016/S0076-6879(05)09019-1 16793410

[bib56] Suzuki SK, Kelley JB, Elston TC, Dohlman HG (2021) Gradient tracking by yeast GPCRs in a microfluidics chamber. Methods Mol Biol 2268: 275–287. 10.1007/978-1-0716-1221-7_18 34085275 PMC8188611

[bib57] Wang Y, Dohlman HG (2004) Pheromone signaling mechanisms in yeast: A prototypical sex machine. Science 306: 1508–1509. 10.1126/science.1104568 15567849

[bib58] Wang Y, Marotti LA, Jr, Lee MJ, Dohlman HG (2005) Differential regulation of G protein alpha subunit trafficking by mono- and polyubiquitination. J Biol Chem 280: 284–291. 10.1074/jbc.m411624200 15519996

[bib59] Yu H, Braun P, Yildirim MA, Lemmens I, Venkatesan K, Sahalie J, Hirozane-Kishikawa T, Gebreab F, Li N, Simonis N, (2008) High-quality binary protein interaction map of the yeast interactome network. Science 322: 104–110. 10.1126/science.1158684 18719252 PMC2746753

