# Supplementary material for: Phosphorylation of RGS regulates MAP kinase localization and promotes completion of cytokinesis
Source: Life Sci Alliance. 2022 Aug 19;5(10):e202101245. doi: 10.26508/lsa.202101245 (PMC9394524; doi:10.26508/lsa.202101245)
Supplement: Supplementary file 1 [file LSA-2021-01245_TableS1.docx]

| Strain | Parent | description |
| --- | --- | --- |
| BY4741 |  | *Mata leu2Δ met15Δ his3Δ ura3Δ* |
| SST2-GFP | BY4741 | *SST2-GFP::HIS3* |
| SST2-GFP BEM1-RUBY2 | BY4741 | *SST2-GFP::HIS3 BEM1-RUBY2::KanMX4* |
| SST2^S539A^ | BY4741 | *sst2^S539A^* |
| SST2^S539D^ | BY4741 | *sst2^S539D^* |
| SST2^S539A^-GFP BEM1-RUBY2 | BY4741 | *sst2^S539A^-GFP::URA3 BEM1-RUBY2::KanMX4* |
| SST2^S539D^-GFP | BY4741 | *sst2^S539D^-GFP::HIS3* |
| SST2^S539D^-GFP BEM1-RUBY2 | BY4741 | *sst2^S539D^-GFP::URA3 BEM1-RUBY2::LEU2* |
| KEL1Δ | BY4741 | *kel1Δ::KanxMX4* |
| GPA1^G302S^ | BY4741 | *gpa1^G302S^::URA3* |
| GPA1^G302S^ SST2-GFP BEM1-RUBY2 | BY4741 | *gpa1^G302S^::URA3 SST-GFP::HIS3 Bem1-RUBY2::LEU2* |
| GPA1^EE^ SST2-GFP BEM1-RUBY2 | BY4741 | *gpa1EE::URA3 SST2-GFP::HIS3 Bem1-RUBY2::LEU2* |
| GPA1^EE^ FUS3-GFP BEM1-RUBY2 | BY4741 | *gpa1EE::URA3 FUS3-GFP::HIS3 Bem1-RUBY2::LEU2* |
| BNI1Δ | BY4741 | *bni1Δ::KanMX4* |
| BNR1Δ | BY4741 | *bnr1Δ::KanMX4* |
| FUS3-GFP BEM1-RUBY2 | BY4741 | *FUS3-GFP::HIS3 BEM1-RUBY2::LEU2* |
| SST2^S539A^ FUS3-GFP BEM1-RUBY2 | BY4741 | *sst2^S539A^ FUS3-GFP::HIS3 BEM1-RUBY2::LEU2* |
| SST2^S539D^ FUS3-GFP BEM1-RUBY2 | BY4741 | *sst2^S539D^ FUS3-GFP::HIS3 BEM1-RUBY2::LEU2* |
| Kel1-gfp | BY4741 | *KEL1-GFP::HIS3* |

**Table S1.**
