# Supplementary material for: Phosphorylation of RGS regulates MAP kinase localization and promotes completion of cytokinesis
Source: Life Sci Alliance. 2022 Aug 19;5(10):e202101245. doi: 10.26508/lsa.202101245 (PMC9394524; doi:10.26508/lsa.202101245)
Supplement: Supplementary file 2 [file LSA-2021-01245_TableS2.docx]

**Table S2.**

| Plasmid | Vector | Description |
| --- | --- | --- |
| pRSII405-Bem1-Ruby2 | pRSII405 | Integrating BEM1-RUBY::LEU2 Vector |
| pRSII406-gpa1^G302S^ | pRSII406 | Integrating gpa1^G302S^::URA3 Vector |
| pRSII406-sst2-GFP | pRSII406 | Integrating SST2-GFP::URA3 Vector |
| PRSII406-sst2^S539A^-GFP | pRSII406 | Integrating sst2^S539A^-GFP::URA3 Vector |
| PRSII416-PADH-SST2-3XFlag | pRSII406 | ADH1 promoter driven overexpression of Sst2-3xFlag |
| PRSII416-PADH-SST2-S539A-3XFlag | pRSII416 | ADH1 promoter driven overexpression of Sst2(S539A)-3xFlag |
| PRSII416-PADH-SST2-S539D-3XFlag | pRSII416 | ADH1 promoter driven overexpression of Sst2(S539D)-3xFlag |
| PRSII416-PADH-Kel1-3xflag | pRSII416 | ADH1 promoter driven overexpression of Kel1-3xFlag |
