# Supplementary material for: Phosphorylation of RGS regulates MAP kinase localization and promotes completion of cytokinesis
Source: Life Sci Alliance. 2022 Aug 19;5(10):e202101245. doi: 10.26508/lsa.202101245 (PMC9394524; doi:10.26508/lsa.202101245)
Supplement: Supplementary file 3 [file LSA-2021-01245_TableS3.docx]

**Table S3.**

| OligoNucleotide  Name | Sequence | gENE | dESCRIPTION |
| --- | --- | --- | --- |
| AHM-22 | GATGCGGTTTTTTACAGGGC | FUS3 | Forward primer for amplifying FUS3 |
| AHM-23 | ATGGATCACCCCTTGTGGTTCT | FUS3 | Reverse primer for amplifying FUS3 |
| AHM-26 | TCTACAGAACGAAGAGGCCAATGATGTCATC |  | Forward primer for mutagenesis for GPA1 EE |
| AHM-27 | AAAGGATCACTTTCGTCTC |  | Reverse primer for mutagenesis for GPA1 EE, Primer from NEB |
| AHM-32 | AAAGACTACAAGCATTACAGAAAC |  | FP for mutagenesis for GPA1G302S |
| AHM-33 | ATACGGCCCTTCAAAATG |  | RP for mutagenesis of GPA1G302S |
| JKM-16 | CATAATCCAAGCCAAACTGAAAATTTCCGTTCACGATATTGGTGACGGTGCTGGTTTA | BEM1 | Forward primer pFA6a labeling for BEM1 |
| JKM-17 | CAAGTAAAGAAGAAAAATGCTTCGTCTTCTAACACTAGATTCGATGAATTCGAGCTCG | BEM1 | Reverse primer pFA6a labeling for BEM1 |
| JKM-18 | GTCTGCGAATTCGGTGACGGTGCTGGTTTAAT | yomRUBY2 | EcoRI Ruby2 primer for cloning into pRSII405 with Bem1 |
| JKM-19 | CGTAGCTCTAGATTACTTATACAATTCATCCA | yomRUBY2 | XbaI Ruby2 reverse primer for cloning into pRSII405 with Bem1 |
| JKM-20 | CTGAACGGTACCGACAACTTATGTGGGAGAGA | BEM1 | KpnI-Bem1 primer for cloning into pRSII405 |
| JKM-21 | CGTACCGAATTCAATATCGTGAACGGAAATTT | BEM1 |  |
| RTM-5 | CAAAGATGCTAGCGCTTTAATAGAAATCCAAGAAAAGTGCGGTGACGGTGCTGGTTTA | SST2 | Forward primer pFA6a yo-tagging for Sst2 |
| RTM-6 | GTGCAATTGTACCTGAAGATGAGTAAGACTCTCAATGAAATCGATGAATTCGAGCTCG | SST2 | Reverse primer pFA6a yo-tagging for Sst2 |
| SVM-17 | GAACTTTACAACTTGTACCCTTCATCACCT | KEL1 | Deletion cassette (250 bp out) |
| SVM-18 | GCACCGCCCAAATACTGCAATCGGACTATTCTGCG | KEL1 | Deletion cassette (250 bp out)` |
| SVM-21 | ACTGGACGTACGATGGTGTT | KEL1 | Deletion verification cassette (500 bp) |
| SVM-22 | CGAACAGCTTCAACGTACCT | KEL1 | Deletion verification cassette (500 bp) |
| WSM-13 | ATCATGTTGCACCCTCATTC | SST2 | Forward primer SST2 c-terminal tag verification |
| WSM-14 | GAATGAATTTGCGTTCAATC | SST2 | Reverse primer SST2 C-terminal tag verification |
| WSM-21 | TCCATCATAACTTGCGTCAGAATATTTCTGACATCATGTTGCACCCTCATGAGCTCGTTTTCGACACTGG | SST2 | pcore s539 insert |
| WSM-22 | GATGCAGGTGATGGATCGTATAGATTAGTAGGAAAGTGTTCCGATAATGGTCCTTACCATTAAGTTGATC | SST2 | pcore s539 insert |
| WSM-23 | TGCGTCAGAATATTTCTGACATCATGTTGCACCCTCATGCTCCATTATCGGAACACTTTCCTACTAATCTATACGATCCA | SST2 | S539A |
| WSM-24 | TGGATCGTATAGATTAGTAGGAAAGTGTTCCGATAATGGAGCATGAGGGTGCAACATGATGTCAGAAATATTCTGACGCA | SST2 | S539A |
| WSM-28 | ATGCATGGATCCGTGCTTATAACTTTAAGAAAAACCAGCGTC | SST2 | With Kpn1 cut-site for creation of integrating vector |
| WSM-29 | ATGCATGGTACCGCCGGTAGAGGTGTGGTCAATAA | SST2 | With BamHI cut-site for creation of integrating vector |
| WSM-37 | GCACCCTCATGCTCCATTATCGG | SST2 | Creation of S539A |
| WSM-38 | AACATGATGTCAGAAATATTCTGACG | SST2 | Creation of S539A |
| WSM-44 | ACACTGAGATTATAGTCCAG | SST2 | Verify Sst2 Integration Vector, Binds upstream of sst2 |
| WSM-45 | TACTATACCTGAGAAAGCAA | SST2 | Verify Sst2 Integration Vector, Binds Downstream of GFP on vector sequence |
| WSM-46 | TGCGTCAGAATATTTCTGACATCATGTTGCACCCTCATGATCCATTATCGGAACACTTTCCTACTAATC | SST2 | Creation of Sst2 S539D from pCORE KO |
| WSM-46 | TGGATCGTATAGATTAGTAGGAAAGTGTTCCGATAATGGATCATGAGGGTGCAACATGATGTCAGAA | SST2 | Creation of Sst2 S539D from pCORE KO |
| WSM-52 | GTACTCAGAGCCACAAGAAA | BNR1 | amplify bnr1 del insert |
| WSM-53 | CCCGATGAACTCATTGAGAA | BNR1 | verify bnr1 deleted |
| WSM-54 | CTAGCGTTCAATTGCCTTCT | BNR1 | verify bnr1 deleted |
| WSM-55 | CTGACGGCTGTGTGTTAATT | BNI1 | amplify bni1 del insert |
| WSM-56 | AGCGAACGCGAAATACAAGT | BNI1 | amplify bni1 del insert |
| WSM-57 | CCAAATCCTTGCTCAACTCT | BNI1 | verify bni1 deleted |
| WSM-66 | GAAAGACCTCAAGAAACTCATTTGGAACGAAATATTTAGTGGTGACGGTGCTGGTTTA | FUS3 | pFA6a tagging |
| WSM-67 | TACATTGTTCTTCGGGTTGATATTTTAATGATAATGATGGCGATGAATTCGAGCTCG | FUS3 | pFA6a tagging |
| JKM-56 | tcgaggtcgacggtatcgatAAGGTGAGACGCGCATAAC | ADH1 | ADH1 promoter amplification forward primer for pRSII416 PADH-Sst2-3xFlag |
| JKM-66 | tatccaccatTGTATATGAGATAGTTGATTGTATGCTTG | ADH1 | AHD1 promoter amplification reverse primer for pRSII416 PADH-Sst2-3xFlag |
| JKM-67 | ctcatatacaATGGTGGATAAAAATAGGAC | SST2 | Sst2 gene forward for pRSII416 PADH-Sst2-3xFlag |
| JKM-68 | ctttgtagtcGCACTTTTCTTGGATTTC | SST2 | Sst2 gene reverse for pRSII416 PADH-Sst2-3xFlag |
| JKM-69 | agaaaagtgcGACTACAAAGACCATGAC | 3xFlag | 3xFlag forward for pRSII416 PADH-Sst2-3xFlag |
| JKM-61 | gcggccgctctagaactagtCTACTTGTCATCGTCATC | 3xFlag | 3xFlag reverse for pRSII416 PADH-Sst2-3xFlag |
| JKM-54 | GACTACAAAGACCATGACGGTGATTATAAAGATCATGACATCGACTACAAGGATGACGATGACAAGTAG | 3xFlag | 3xFlag sequence for annealing, forward |
| JKM-55 | CTACTTGTCATCGTCATCCTTGTAGTCGATGTCATGATCTTTATAATCACCGTCATGGTCTTTGTAGTC | 3xFlag | 3xFlag sequence for annealing, reverse |
| CJM-64 | ggcgaattgggtaccgggccAAGGTGAGACGCGCATAAC | ADH1 | ADH1 promoter amplification forward primer for pRSII416 PADH-Kel1-3xFlag |
| CJM-65 | atccagccatTGTATATGAGATAGTTGATTGTATGCTTG | ADH1 | ADH1 promoter amplification reverse primer for pRSII416 PADH-Kel1-3xFlag |
| CJM-66 | ctcatatacaATGGCTGGATTCAGCTTC | KEL1 | Kel1 amplification forward primer pRSII416 PADH-Kel1-3xFlag |
| CJM-67 | ctttgtagtcTAGTAGATCGCTGTCAGC | KEL1 | Kel1 amplification reverse primer pRSII416 PADH-Kel1-3xFlag |
| CJM-68 | cgatctactaGACTACAAAGACCATGAC | 3xFlag | 3xFlag forward for pRSII416 PADH-Kel1-3xFlag |
| CJM-69 | agggaacaaaagctggagctCTACTTGTCATCGTCATC | 3xFlag | 3xFlag reverse for pRSII416 PADH-Kel1-3xFlag |
